# Action mechanism of snake venom l-amino acid oxidase and its double-edged sword effect on cancer treatment: Role of pannexin 1-mediated interleukin-6 expression

**DOI:** 10.1016/j.redox.2023.102791

**Published:** 2023-06-22

**Authors:** Nam V. Truong, Trinh T.T. Phan, Tzu-Sheng Hsu, Phan Phu Duc, Lih-Yuan Lin, Wen-Guey Wu

**Affiliations:** a Institute of Bioinformatics and Structural Biology, College of Life Science, National Tsing Hua University, Hsinchu, 300044, Taiwan, ROC; b Institute of Molecular and Cellular Biology, College of Life Science, National Tsing Hua University, Hsinchu, 300044, Taiwan, ROC

**Keywords:** L-amino acid oxidase, Oxidative stress, N-linked glycans, Interleukin-6, Pannexin 1, Metastasis

## Abstract

Snake venom l-amino acid oxidases (svLAAOs) have been recognized as promising candidates for anticancer therapeutics. However, multiple aspects of their catalytic mechanism and the overall responses of cancer cells to these redox enzymes remain ambiguous. Here, we present an analysis of the phylogenetic relationships and active site-related residues among svLAAOs and reveal that the previously proposed critical catalytic residue His 223 is highly conserved in the viperid but not the elapid svLAAO clade. To gain further insight into the action mechanism of the elapid svLAAOs, we purify and characterize the structural, biochemical, and anticancer therapeutic potentials of the Thailand elapid snake *Naja kaouthia* LAAO (NK-LAAO). We find that NK-LAAO, with Ser 223, exhibits high catalytic activity toward hydrophobic l-amino acid substrates. Moreover, NK-LAAO induces substantial oxidative stress-mediated cytotoxicity with the magnitude relying on both the levels of extracellular hydrogen peroxide (H_2_O_2_) and intracellular reactive oxygen species (ROS) generated during the enzymatic redox reactions, but not being influenced by the N-linked glycans on its surface. Unexpectedly, we discover a tolerant mechanism deployed by cancer cells to dampen the anticancer activities of NK-LAAO. NK-LAAO treatment amplifies interleukin (IL)-6 expression via the pannexin 1 (Panx1)-directed intracellular calcium (iCa^2+^) signaling pathway to confer adaptive and aggressive phenotypes on cancer cells. Accordingly, IL-6 silencing renders cancer cells vulnerable to NK-LAAO-induced oxidative stress together with abrogating NK-LAAO-stimulated metastatic acquisition. Collectively, our study urges caution when using svLAAOs in cancer treatment and identifies the Panx1/iCa^2+^/IL-6 axis as a therapeutic target for improving the effectiveness of svLAAOs-based anticancer therapies.

## Highlights

• His 223 residue is highly conserved in the viperid but not the elapid svLAAO clade.
• Activity and cytotoxicity of the elapid NK-LAAO are independent of N-linked glycans.
• NK-LAAO treatment enables cancer cells to produce IL-6 via the Panx1/iCa^2+^ pathway.
• IL-6 confers adaptive and metastatic phenotypes on NK-LAAO-treated cancer cells.
• IL-6 silencing heightens NK-LAAO-induced oxidative stress and anticancer activities.

## Graphical abstract

**Figure:**
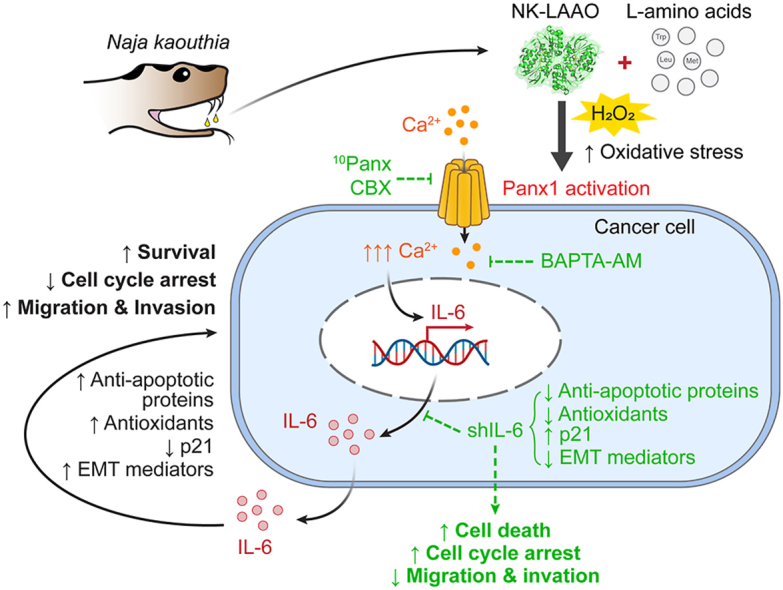

## 1 Introduction

l-amino acid oxidases (LAAOs, EC 1.4.3.2) are dimeric glycosylated flavoenzymes found in diverse organisms [1,2]. The LAAOs from snake venom (svLAAOs) are among the most studied LAAOs and the main components constituting snake venom cocktails [3,4]. svLAAOs catalyze the stereospecific oxidative deamination of l-amino acid to α-keto acid along with generating ammonia (NH_3_) and hydrogen peroxide (H_2_O_2_) [2,4]. Of them, H_2_O_2_ is suggested as the major cytotoxic contribution of the enzyme to host cells [[5], [6], [7], [8]]. In addition, glycan moieties on svLAAOs are implicated in anchoring the enzyme to the cell surface for locally delivering H_2_O_2_ into the cell, leading to cell apoptosis [9,10]. Early three-dimensional (3-D) structural studies of the viperid *Calloselasma rhodostoma* LAAO (CR-LAAO) [2,4] suggested that the His residue at position 223 (His223) may act as a catalytic base to abstract a proton from the α-amino group (α-NH_3_^+^) of amino acid substrates in the zwitterion form, whereas experimental data from a subsequent study [11] indicated that His223 is not essential for the catalytic activity of the viperid *Daboia russelii* LAAO (DR-LAAO). To date, the proposed catalytic role for His223 has still been widely recognized as a general catalytic mechanism for svLAAOs [[12], [13], [14], [15]]. Accordingly, it is important to affirm the roles of the active site-related residue His223 in the enzymatic activity of svLAAOs as well as the N-linked glycans on the surface of svLAAOs in their action toward host cells.

svLAAOs exhibit strong cytotoxic activities driven by the enzyme-produced H_2_O_2_ against multiple cancer cell lines [[5], [6], [7], [8],^10^,[16], [17], [18]]. Moreover, the delivery of LAAOs as well as H_2_O_2_-producing enzymes encapsulated in bulk nanovesicles effectively induces cancer cell death both *in vitro* and *in vivo*, suggesting that the H_2_O_2_-generating svLAAOs may serve as potential anticancer agents [19,20]. Unexpectedly, in addition to triggering cell death, H_2_O_2_ renders cancer cells resistant to reactive oxygen species (ROS)-based anticancer therapies by promoting cell proliferation and metastasis [21,22]. Thus, whether svLAAOs, whose anticancer activities primarily rely on the production of H_2_O_2_, can be clinically used in cancer treatment requires further investigation.

Interleukin 6 (IL-6) is a pleiotropic secreted cytokine produced by many cell types, including immune, stromal, and cancer cells, and regulates cellular responses to stressors [23,24]. In cancer, IL-6 can act in both autocrine and paracrine manners to direct the expression of downstream target molecules associated with cancer cell survival, proliferation, metastasis, and drug resistance [[25], [26], [27], [28], [29]]. IL-6 stimulates the epithelial‐to‐mesenchymal transition (EMT) process of tumor cells, contributing to the malignant progression of tumors in the late stages of cancer development [25,30]. Nonetheless, how IL-6 influences the responses of cancer cells to svLAAOs has not been addressed.

Human pannexin 1 (Panx1) is a non-selective large-pored channel formed by seven subunits [[31], [32], [33]]. Activated Panx1 channels allow the passages of calcium (Ca^2+^), adenosine triphosphate (ATP) and its degradation products (ADP, AMP, and adenosine), and other molecules with molecular mass up to 1 kDa [[34], [35], [36]]. Panx1 channels localized on plasma and endoplasmic reticulum (ER) membranes contribute to regulating the intracellular Ca^2+^ (iCa^2+^) levels, via several mechanisms, including the intercellular Ca^2+^ diffusion between adjacent cells, the influx of Ca^2+^ from the extracellular environment, and/or the release of Ca^2+^ from the ER lumen [32,33,[36], [37], [38]]. Uncontrolled activation of Panx1 channels and/or aberrant iCa^2+^ levels result in enhanced metastasis and drug tolerance of cancer cells [[39], [40], [41]]. However, the molecular mechanisms underlying these effects of Panx1 as well as links among svLAAOs, IL-6, and the Panx1/iCa^2+^ signaling have not been investigated.

In this study, we investigate the phylogenetic relationships and active site-related residues among the redox enzymes svLAAOs. We used the purified elapid snake *Naja kaouthia* LAAO (NK-LAAO) as a representative to understand the action mechanism and anticancer potentials of svLAAOs. We unravel that although this enzyme shows substantial oxidative stress-mediated cancer cell death, it may be a double-edged sword in cancer treatment. NK-LAAO treatment activates the Panx1/iCa^2+^ signaling pathway to potentiate cancer cells to induce IL-6 expression, leading to cytotoxic tolerance and acquisition of the metastatic phenotype. Our findings suggest that targeting IL-6 and its regulatory pathway may enhance the responsiveness of cancer cells to svLAAOs-based anticancer therapies.

## 2 Materials and methods

### 2.1 Venom, regents, chemicals, and materials

The Thailand *Naja kaouthia* venom lyophilized was obtained from the venom supplier Latoxan (Valence, France). Reagents for cell cultures were purchased from Invitrogen Gibco (Grand Island, NY, USA). Other reagents, chemicals, and materials used in this study were purchased from Sigma-Aldrich (St. Louis, MO, USA) unless specifically stated. TRIzol and the chloroform replacement reagent used for extracting total RNA were purchased from Invitrogen Ambion (Carlsbad, CA, USA) and Cyrusbioscience (Taipei, Taiwan), respectively. The RevertAid First Strand cDNA Synthesis Kit was from Thermo Scientific (Thermo Fisher Scientific Inc., Vilnius, Lithuania). Primers and the Power SYBR Green PCR Master Mix used for qRT-PCR assays were purchased from Integrated DNA Technologies (Coralville, IA, USA) and Applied Biosystems (Thermo Fisher Scientific Inc., Woolston, Warrington, UK), respectively. The fluorescent dyes 2,7-dichlorofluorescin diacetate (DCFH-DA) and Fluo-4 AM used to detect intracellular reactive oxygen species (ROS) and calcium concentrations were obtained from Calbiochem/Merck (Darmstadt, Germany) and Invitrogen (Thermo Fisher Scientific Inc., Eugene, OR, USA), respectively. The intracellular calcium chelator BAPTA-AM was purchased from Enzo Life Sciences (Farmingdale, NY, USA). The pannexin 1 mimetic inhibitory peptide ^10^Panx was obtained from Tocris (Tocris Bioscience, Bristol, UK). Matrigel matrix for transwell cell invasion assays was obtained from Corning (Bedford, MA, USA).

### 2.2 Purification of native and deglycosylated NK-LAAOs

The native NK-LAAO was purified on a Fast protein liquid chromatography (FPLC) system (AKTA Purifier 10 System; GE Healthcare Life Science, Marlborough, MA, USA) through three sequential columns (Figs. S1A–C) and the protein concentration was monitored by absorbance at a wavelength of 280 nm. Firstly, *Naja kaouthia* venom powder was dissolved in 50 mM sodium phosphate buffer containing 200 mM NaCl (pH 6.2) and centrifuged at 10,000×*g* for 10 min at 4 °C to discard the precipitates before being passed through a Superdex 75 Increase 10/300 GL column (Cytiva, Marlborough, MA, USA), which separate molecules based on their sizes and molecular weights (Fig. S1A). The column was equilibrated and eluted by two column volumes (CV) (each CV equal to 24 ml) of the same buffer as mentioned above with a flow rate of 1 ml per min. The first fraction containing native NK-LAAO was then collected and applied to the next, HiTrap Q HP 5 ml (Cytiva, Marlborough, MA, USA), an anion exchange chromatography column (Fig. S1B). The HiTrap Q column was equilibrated by 3 CV of buffer A (50 mM Tris-HCl, pH 8.0) and proteins were eluted by 15 CV of buffer B (50 mM Tris-HCl containing 1 M NaCl, pH 8.0) with a linear gradient from 0 to 100% (v/v) and flow rate of 0.5 ml per min. The NK-LAAO containing fraction 2 eluted by between 25 and 30% (v/v) buffer B from the HiTrap Q column was applied to the next, Heparin HP 5 ml (Cytiva, Marlborough, MA, USA), an affinity column (Fig. S1C). Similarly, the Heparin column was also equilibrated by 3 CV of buffer A (50 mM sodium phosphate, pH 7.4) and proteins were eluted by a total of 15 CV of buffer B (50 mM sodium phosphate containing 1 M NaCl, pH 7.4), 10 CV for the linear gradient from 0 to 50% (v/v) B followed by 5 CV for the linear gradient up to 100% (v/v) B. The flow rate was set up at 0.5 ml per min. The purified native NK-LAAO was collected from fraction 3 with the elution gradient from 25 to 30% (v/v) buffer B. Noticeably, samples were always desalted, exchanged to the suitable buffers (buffer A of the next column), and concentrated prior to being loaded onto ion exchange and affinity columns.

For deglycosylation experiments, the reaction mixture (5 ml) containing the purified native NK-LAAO (0.39 mg) and the recombinant endoglycosidase F3 (endo F3) (0.39 mg) in 50 mM sodium phosphate (pH 7.4) was incubated at 37 °C for 2 h, followed by 4 °C overnight, and then was separated by the Superdex 200 Increase 10/300 GL column (GE Healthcare Bio-Sciences AB, Uppsala, Sweden) (Fig. S1D). The deglycosylated NK-LAAO (Degly.NK-LAAO) was collected from the first fraction. For endo F3 expression, the endo F3 encoding gene was inserted into pETM-41 vector (6His-MBP-Tev-EndoF3) (Addgene, Watertown, MA, USA) and expressed in BL21 (DE3) competent cells (Yeastern Biotech, Taipei, Taiwan). Recombinant endo F3 was purified using a Ni-NTA column (Cytiva, Marlborough, MA, USA).

All fractions from each column were analyzed by SDS-PAGE (12% (w/v) acrylamide) under reducing conditions and by enzymatic activity using L-Leu (Sigma-Aldrich) as substrate (data not shown). The native NK-LAAO fraction (fraction 3, Fig. S1C) was analyzed by measuring LAAO activity (Fig. S1E). The purity of native (fraction 3, Fig. S1C) and deglycosylated (fraction 1, Fig. S1D) NK-LAAO was analyzed by SDS-PAGE (Fig. S1F) and mass spectrometry (Fig. S1G) using the matrix-assisted laser desorption/ionization-time of flight (MALDI-TOF) method, which indicated their molecular weights being 61.124 kDa and 59.764 kDa, respectively.

### 2.3 Enzyme activity assay and kinetic study

The LAAO activity was evaluated using a colorimetric assay in a 96-well microplate (PerkinElmer, Waltham, MA, USA), adapted from a previous study [42]. Briefly, 10 μl of enzyme solution was added with 40 μl of l-amino acid substrate (L-Leu) to yield a final reaction mixture of 1 μg of LAAO against 4 mM l-amino acid in 100 mM Tris-HCl, pH 8.5. The mixture was incubated at 25 °C for 30 min prior to being sequentially added with 50 μl of 10 U/ml horseradish peroxidase (HRP) (Thermo Scientific; Thermo Fisher Scientific Inc., Rockford, IL, USA) in 100 mM Tris-HCl (pH 8.5) and 50 μl of 2 mM 3,3A,5,5A-tetramethylbenzidine (TMB) (Acros Organics; Thermo Fisher Scientific Inc., Germany) in dimethyl sulfoxide (DMSO) (Sigma-Aldrich). The reaction was then stopped by being added with 50 μl of 2 M sulfuric acid (H_2_SO_4_) solution and the absorbance of the mixture was recorded at 405 nm by a BioTek 800 TS Absorbance Reader (Agilent, Santa Clara, CA, USA).

For the kinetic study, the steady-state kinetics of the enzyme was firstly determined via the initial velocity of NK-LAAO with different l-amino acid substrates (Sigma-Aldrich) at varying concentrations (L-Leu (0.0625-4 mM), L-Trp (0.03125-1 mM), L-Met (0.03125-4 mM), L-Arg (0.125-4 mM), and L-Glu (0.625-20 mM)) for 2 min (L-Trp, L-Met, and L-Arg), 4 min (L-Leu), or 240 min (L-Glu). Other conditions for the reaction were kept constant as mentioned above. The recorded values of absorbance were used to calculate the number of released H_2_O_2_ per sec (mM/sec) based on the standard curve of H_2_O_2_. The kinetic parameters Km and kcat were calculated by fitting the Michaelis-Menten equation to initial velocity data using nonlinear-regression analysis in the GraphPad Prism 7 software (GraphPad Inc., San Diego, CA, USA).

### 2.4 Sequence alignments and phylogenetic reconstruction

The protein sequence of the elapid NK-LAAO was obtained from the venom gland transcriptomes of Tan et al. [43]. Other 37 LAAO protein sequences (36 from snakes and 1 from a lizard) were retrieved from the protein database of non-redundant protein sequences (nr) (NCBI) using blastp (protein-protein BLAST) program (https://blast.ncbi.nlm.nih.gov/Blast.cgi) and cross-referenced with UniProtKB database (https://www.uniprot.org/). The blastp results were filtered by setting up the parameters of E-value close to zero (10^−5^), percentage of identity greater than 30%, and query coverage greater than 90% to get homologs of NK-LAAO [44]. Signal peptides of all LAAO protein sequences either experimentally annotated or predicted by using the SignalP 6.0 server (https://services.healthtech.dtu.dk/service.php?SignalP) were removed from mature forms [45]. A total of 38 representative sequences of LAAOs were used to reconstruct a phylogenetic tree. Of them, 10 and 27 svLAAOs are from Elapidae and Viperidae families, respectively, and one is from the lizard species (*Varanus komodoensis*) used as an outgroup (Table S1).

The multiple alignments of 38 LAAO protein sequences were carried out on the Multiple Sequence Comparison by Log-Expectation (MUSCLE) program integrated into the Molecular Evolutionary Genetics Analysis (MEGA) software version 11 (M11) with default parameters [46]. The phylogenetic reconstruction was inferred by using the Maximum Likelihood (ML) method and the best model of JTT + G substitution (5 rate categories) with 1000 bootstrap replications for the tree topology [47]. The output tree with the highest log likelihood (−7916.74) was rooted using the outgroup and displayed with iTOL [48]. There was a total of 579 positions in the final dataset of 38 amino acid sequences. The tree was drawn to scale, with branch lengths proportional to the number of amino acid substitutions per site (a scale bar of 0.1 was shown).

### 2.5 3-D structural modeling

A homology modeling of NK-LAAO was performed on the SWISS-MODEL server (https://swissmodel.expasy.org/interactive#structure) [49] using an X-ray crystal structure of the elapid *Naja atra* (NA)-LAAO (PDB ID: 5Z2G) as a template. The output model was then applied for energy minimizations using the YASARA Energy Minimization Server (http://www.yasara.org/minimizationserver.htm) [50] followed by PHENIX geometry minimization (iterations = 5 and macro cycles = 15) with secondary structure restraints [51]. The stereochemical quality of the refined model was evaluated by the ERRAT [52] and PROCHECK programs [53], included in the SAVES v6.0 webserver (https://saves.mbi.ucla.edu/), and the MolProbity webserver (http://molprobity.biochem.duke.edu/) [54].

For molecular docking, only chain A with its bound flavin adenine dinucleotide (FAD) of the refined NK-LAAO model (NK-LAAO-A) was used as a receptor, which was subsequently pretreated by adding the nonpolar hydrogen and Kollman charges and assigned AD4 type. The 3-D structure of the L-Trp (TRP) ligand was obtained from the RCSB PDB database at https://www.rcsb.org/and pretreated by adding hydrogens and optimizing geometry using Avogadro (version 1.2.0) software [55] and saved in MOL2 format. The docking model of the NK-LAAO-A with TRP was performed by the Autodock4 (version 4.2.6) program in the AutoDockTools (version 1.5.7) software [56]. The NK-LAAO-A residues were kept rigid while the ligand TRP was completely flexible with five rotatable bonds. The searching space for binding sites of TRP was set with grid box center (x = −59.361, y = 13.150, z = −11.269) with 0.525 Å spacing and gride box size (x = 50, y = 50, z = 50). The docking was carried out using the Lamarckian genetic algorithm (LGA) with 50 runs and 25,000,000 energy evaluations. Other docking parameters were set as default. The best binding mode with the free energy of binding of −5.28 kcal/mol was chosen for further analysis. Molecular graphics images were generated using PyMOL [57] and LIGPLOT [58]. The schematic diagram of protein domain structures was generated by DOG [59].

The N-linked glycosylation sites were predicted with the NetNGlyc-1.0 web server (https://services.healthtech.dtu.dk/service.php?NetNGlyc-1.0) [60].

### 2.6 Cell culture and treatments

The human lung adenocarcinoma cell line A549 obtained from the American Type Culture Collection (ATCC, Manassas, VA, USA) was maintained and cultured as previously described [61]. In brief, cells were cultivated in Roswell Park Memorial Institute (RPMI) 1640 medium supplemented with l-glutamine (2 mM), heat-inactivated fetal bovine serum (FBS; 10% (v/v)), sodium bicarbonate (0.22% (w/v)), and the antibiotics penicillin (100 units/mL) and streptomycin (100 μg/mL) in a humidified incubator at 37 °C and 5% (v/v) CO_2_. Cells were seeded at densities of 8 × 10^3^ cells per well in 100 μl (96-well plate), 3.5 × 10^4^ cells per well in 0.3 ml (24-well plate), 1.2 × 10^5^ cells per well in 1.4 ml (6-well plate), 2.5 × 10^5^ cells per 6-cm dish in 2.4 ml, and 7 × 10^5^ cells per 10-cm dish in 10 ml of medium except for special cases stated. Cells were pre-incubated for 24 h or 48 h for stable attachment and reaching the desired confluence of 70-80% before being treated with appropriate reagents. Cells were counted by a hemocytometer and only cells between passages (P)2 and P5 were used for conducting experiments.

Following initial incubation for cell attachment, the culture medium was removed and replaced with a fresh medium containing appropriate treatment reagents. All treatment reagents were added to the culture medium to reach predesigned concentrations immediately before cell treatments and the medium was then thoroughly mixed using a vortex. l-amino acids, catalase (CAT), N-acetylcysteine (NAC), carbenoxolone (CBX), ATP, and apyrase were dissolved in double-distilled water (ddH_2_O). ^10^Panx and BAPTA-AM were dissolved in DMSO. l-amino acids, CAT, or apyrase were pre-mixed in the culture medium together with NK-LAAO immediately before the medium was applied to the cells. NAC, ^10^Panx, or CBX were pretreated for 1 h and BAPTA-AM was pretreated for 30 min before the cells were treated with NK-LAAO.

### 2.7 Cell viability assay

Cell viability was evaluated by using a colorimetric MTT (3-(4,5-dimethylthiazol-2-yl)-2,5-diphenyltetrazolium bromide) assay. Briefly, cells were seeded in 96-well plates (TPP Techno Plastic Products AG, Trasadingen, Switzerland) and pre-incubated for 24 h. The culture medium was then discarded from each well and replaced with fresh medium containing the indicated concentrations of treatment reagents. After 24 h incubation, the medium was replaced with medium containing MTT (Sigma-Aldrich; 0.4 mg/ml) and cells were incubated for an additional 2.5 h. The supernatant was subsequently removed, and formazan crystals were dissolved in DMSO (200 μl each well) followed by being incubated at 37 °C for 45 min. Absorbance at 570 with a reference wavelength of 650 nm was measured using a BioTek 800 TS Absorbance Reader (Agilent, Santa Clara, CA, USA). Cells treated with vehicles served as controls and MTT must be protected from light. The half-maximal inhibitory concentration (IC50) value (the concentration of NK-LAAO at which it inhibits cell viability by 50%) was calculated by the GraphPad Prism 7 software using a non-linear regression model based on four parameters logistic equation to fit the dose-response data into a curve.

### 2.8 Enzyme-linked immunosorbent assay (ELISA)

A549 cell culture supernatants were collected from 24-well plates for the quantification of IL-6 protein levels. ELISA was performed using a human IL-6 Uncoated ELISA kit (Invitrogen, Campus Vienna Biocenter 2, Vienna, Austria) according to the manufacturer's instructions. The IL-6 concentrations of samples were interpolated from a IL-6 standard curves (parabolic curves), which were fitted using a second order polynomial, and corrected for sample dilution.

### 2.9 Flow cytometric analysis

Cells used for flow cytometric experiments were cultured in 6-cm dishes (Corning Inc., Durham, NC, USA). A total of 10,000 events per sample was analyzed after excluding debris, cell death, or clumps via a proper gating of forwarding and side scatters. The raw data collected from the BD Accuri C6 flow cytometer (BD Biosciences, San Jose, CA, USA) were further analyzed using FlowJo v7.6.1 software (FlowJo LLC, Ashland, OR, USA). A representative histogram was shown concomitantly with the quantification of the mean fluorescence intensity (MFI) between treated and untreated samples from three independent experiments. All experiments using fluorescent reagents were protected from light.

For measurement of cell cycle phase distribution, cells were treated with appropriate reagents for 24 h before the medium was removed and washed twice with warm phosphate-buffered saline (PBS). The cells were then de-attached by trypsin at 37 °C for 5 min and resuspended in fresh medium in 1.5 ml microcentrifuge tubes. After being centrifuged at 700×*g* for 5 min, the cells were fixed in cold ethanol (70% (v/v)) and stored at 4 °C overnight. The cells were then collected via centrifugation (700×*g* for 5 min) and stained with a staining solution containing 5 μg/ml propidium iodide (PI) (Sigma-Aldrich) and 100 μg/ml Ribonuclease A (RNase A) (Sigma-Aldrich) at 37 °C for 30 min. Fluorescence was measured at 488/585 nm (FL2-A channel) excitation/emission wavelengths.

For measurement of intracellular ROS, two fluorescent probes, including Dihydroethidium (DHE; Sigma-Aldrich) and DCFH-DA (Calbiochem/Merck, Darmstadt, Germany), were used to assess general ROS production [62]. Cells receiving appropriate treatments for 24 h were added with either DHE or DCFH-DA to reach a final concentration of 1 μM or 5 μM, respectively, and incubated at 37 °C for 30 min. The cells were then harvested in PBS in 1.5 ml microcentrifuge tubes for subsequent fluorescent measurements at 488/585 nm (FL2-A channel) or 488/530 nm (FL1-A channel) excitation/emission wavelengths, respectively.

For measurement of intracellular calcium (iCa^2+^), cells were collected, resuspended in the medium containing 2 μM Fluo-4 AM (Invitrogen; Thermo Fisher Scientific Inc., Eugene, OR, USA), and incubated at 37 °C for 30 min. After incubation, the Fluo-4 AM-containing medium was removed by centrifugation (700×*g* for 5 min) and the cell pellet was re-suspended in PBS. Cells were then incubated at 37 °C for an additional 30 min prior to being subjected to flow cytometric analysis of Fluo-4 fluorescence (FL1-A channel; 488/530 nm wavelengths).

### 2.10 Caspase-3 activity assay

Cells were collected and lysed with a lysis buffer (1% Triton™ X-100, 1% NP-40, 2 μg/ml aprotinin, 2 μg/ml leupeptin, and 2 mM PMSF), and then placed on ice for 10 min. The supernatants were collected by centrifugation at 16,000×*g* for 30 min at 4 °C and the protein concentrations were measured using the Bio-Rad protein assay (Bio-Rad, Hercules, CA, USA). Caspase-3 activity assays were carried out using 96-well plates. Each reaction mixture (100 μl) contained 50 μg of protein sample and 50 μM Ac-DEVD-AFC substrate in reaction buffer (10 mM HEPES, 2 mM EDTA, 10 mM KCl, 1.5 mM MgCl_2_, and 10 mM DTT). The mixtures were incubated at 37 °C for 1 h before fluorescence was measured at 405/495 nm excitation/emission wavelengths using a VICTOR Nivo Multimode Plate Reader (PerkinElmer Inc., Shelton, CT, USA).

### 2.11 Cell morphology analysis

A549 cells were seeded in 6-cm dishes at a density of 5 × 10^4^ cells per dish and pre-incubated for 48 h before being treated with the indicated NK-LAAO concentrations. The cell morphology was captured at 24 h and 48 h post treatments using a Nikon TMS-F Inverted Phase Contrast Microscope (Tokyo, Japan) at 100× magnification coupled with a Dino-Eye AM423X Digital Microscope Eyepiece Camera USB 2.0 (AnMo Electronics Corporation, New Taipei City, Taiwan).

### 2.12 Luminescent assay for totally extracellular ATP measurement

Extracellular adenosine triphosphate (eATP) was measured via quantification of luminescence from a luciferase/D-luciferin reaction in the presence of ATP. The assay was performed using an ATP Kit SL (BioThema, Handen, Sweden) based on the manufacturer's protocol with slight modifications. In principle, cells were seeded into a 96-well white polystyrene microplate (Thermo Scientific; Thermo Fisher Scientific Inc., Roskilde, Denmark) and cultured for 24 h to allow cell attachment. The medium in each well was then replaced with 60 μl of fresh medium containing a defined concentration of treatment reagents, and cells were incubated for an additional 24 h. After 24 h of treatments, cells were sequentially added with 60 μl of Tris-EDTA buffer (pH 7.75) and 20 μl of the luciferase/D-luciferin mixture, and the luminescence corresponding to sample ATP (L_sam_) was measured with a VICTOR3 Multilabel Plate Reader (PerkinElmer Inc., Shelton, CT, USA). Subsequently, 5 μl of a 2 μM internal ATP standard was added into each well and the light emission corresponding to the sample and standard ATP (L_sam + std_) was measured for calibration. The sample ATP concentration (eATP) in each well was determined following equation: eATP = 71.4 × L_sam_/(L_sam + std_ - L_sam_). Of which, 71.4 (nM) is the concentration of ATP standard in each well ((5 μl × 2 × 10^3^ nM)/140 μl).

### 2.13 Generation of stable knockdown cell lines

The lentiviral short hairpin RNA (shRNA) constructs pLKO.1-shIL-6 #1 (TRCN0000059207) and pLKO.1-shIL-6 #2 (TRCN0000059206) used to express specific shRNAs (shIL-6 #1 and shIL-6 #2, respectively) against human IL-6 or the lentiviral non-silencing shRNA construct pLKO.1-scramble (ASN0000000004) used as a negative control was purchased from the RNA Technology Platform and Gene Manipulation Core at the Institute of Molecular Biology, Academia Sinica (Taipei, Taiwan). Detailed information on the targets and oligo sequences of constructs is shown in Table S2. Lentiviral particles were made in HEK293T cells by co-transfecting cells with 12 μg of the lentiviral shRNA vector (pLKO.1-shIL-6 #1, pLKO.1-shIL-6 #2, or pLKO.1-scramble), 9 μg of the psPAX2 packaging, and 3.6 μg of the pMD2.G envelope plasmids using Maestrofectin reagent (MaestroGen, Hsinchu City, Taiwan) following the manufacturer's instructions. The supernatant was collected 72 h post-transfection by centrifugation (300×*g* for 5 min) followed by being filtered through a 0.45 μm pore-size polyvinylidene difluoride (PVDF) membrane. The vial supernatant was either immediately used to infect A549 cells or stored at 4 °C (within one week) or −80 °C (several months). For the generation of stably-transduced cell lines, 1 × 10^5^ A549 cells were seeded into 6-well plates (Corning Inc., Wujiang, Jiangsu, China) and grown overnight. After the cells had reached approximately 30-50% confluence, cells were exposed to 1 ml of the lentiviral particles-containing supernatant in the presence of 10 μg/ml Polybrene for 4 h. Subsequently, the medium was replaced with a complete RPMI medium, and the cells were incubated for an additional 20 h before being subcultured. Twenty-four hours after the subculture, the transduced cells started to undergo selection in an RPMI medium containing 1.5 μg/ml puromycin. Cells were subcultured every 2 days into 1.5 μg/ml puromycin-containing medium and this selection process lasted until all non-transduced cells in the control well were dead. The stable knockdown cell lines were subjected to testing for IL-6 gene knockdown efficiency prior to being applied for further experiments.

### 2.14 RNA isolation, reverse transcription, and quantitative real-time polymerase chain reaction (qRT-PCR)

Total RNA was extracted from cells using TRIzol reagent following the manufacturer's protocol. RNA concentration was measured using a NanoDrop 2000 spectrophotometer (Thermo Scientific; Thermo Fisher Scientific Inc., Wilmington, DE, USA) and its purity was assessed via the absorbance ratios A260/A280 and A260/A230. Three μg of isolated RNA were reverse-transcribed into complementary DNA (cDNA) in a final volume of 20 μl using the RevertAid First Strand cDNA Synthesis Kit according to the manufacturer's instructions. A “no amplification control” (sample without reverse transcriptase (RT) enzyme) was included for each sample to detect possible genomic DNA contamination in the RNA preparations. The resultant cDNA was used to evaluate the transcript levels of targeted genes by the qRT-PCR method using the Power SYBR Green PCR Master Mix and pre-designed primers of targeted genes (Table S3). The qRT-PCR reaction was performed on a StepOnePlus Real-Time PCR system (Applied Biosystems; Thermo Fisher Scientific Inc., Foster City, CA, USA) following the thermal cycle conditions of initial denaturation for 10 min at 95 °C followed by 40 cycles of amplification each includes 95 °C for 15 s and 60 °C for 1 min. The mRNA expression levels of the target genes represented by threshold cycle (Ct) values were normalized to those of the housekeeping gene GAPDH (glyceraldehyde 3-phosphate dehydrogenase) and relatively calculated using the 2^−ΔΔCt^ method. The mRNA expression levels of GAPDH were calculated as 2^−ΔCt^.

### 2.15 Cell migration and invasion assays

Transwell migration and invasion assays were performed as previously described [61] with slight modifications using transparent polyethylene terephthalate (PET) membrane inserts (8-μm pore size; Corning Inc., Durham, NC, USA) placed in a 24-well cell culture plate (Corning Inc., Durham, NC, USA). For invasion assays, the inserts were pre-coated with Matrigel basement membrane matrix (Corning Inc., Bedford, MA, USA) prior to cell seeding. The uncoated (for migration assays) or Matrigel-coated (for invasion assays) inserts were loaded with 2.5 or 5 × 10^4^ A549 cells, respectively, resuspended in 200 μl of RPMI medium containing 1% (v/v) FBS in the presence or absence of 0.1 μg/ml NK-LAAO while the bottom wells were filled with 750 μl of a basic RPMI medium containing 10% (v/v) FBS. After 18 h incubation, cells attached to both the upper and lower surface of the transwell membrane were fixed with 4% (w/v) paraformaldehyde (Electron Microscopy Sciences, Hatfield, PA, USA) for 10 min and permeabilized with absolute methanol for 20 min prior to being stained with 0.05% (w/v) crystal violet for 15 min at room temperature. Subsequently, the non-migrated and -invaded cells on the upper surface were gently removed by cotton swabs whereas the migrated and invaded cells on the lower surface were imaged using a Dino-Eye AM423X Digital Microscope Eyepiece Camera USB 2.0 coupled with the Nikon TMS-F Inverted Phase Contrast Microscope. Crystal violet bound to the cells was eluted with 33% (v/v) acetic acid, and absorbance at 595 nm was measured using a microplate reader (Bio-Rad, Hercules, CA, USA).

### 2.16 Western blot analysis

Cells treated with appropriate reagents were washed twice with cold PBS and gently harvested using a cell scraper. 1.3 ml of the suspended cells in cold PBS was transferred to a 1.5 ml microcentrifuge for centrifugation at 700×*g* for 5 min at 4 °C. The supernatant was then removed, and the cell pellet was lysed with the cold RIPA (radioimmunoprecipitation assay) buffer (50 mM Tris-HCl (pH 8.0), 150 mM NaCl, 1% (v/v) NP-40, 0.1% (w/v) SDS, 0.5% (w/v) sodium deoxycholate, and 5 mM EDTA (pH 8.0)) supplemented with the phosphatase (50 mM NaF and 1 mM Na_3_VO_4_) and protease (2 μg/ml Aprotinin, 5 μg/ml Leupeptin, 1 μg/ml Pepstatin, and 1 mM PMSF) inhibitors. The mixture was left on ice for a total of 40 min with vortexing every 10 min before being centrifuged at 16,000×*g* for 20 min at 4 °C. After centrifugation, the cell lysate supernatant was collected, and the protein concentration was determined using the Bio-Rad protein assay (Bio-Rad, Hercules, CA, USA). Cell lysate proteins were separated on a 10% SDS-PAGE and transferred onto a 0.2 μm PVDF membrane (GE Healthcare, Milwaukee, WI, USA) using a transfer cell (Bio-Rad, Hercules, CA, USA). The membrane was blocked with a blocking buffer containing 5% (w/v) skim milk in Tris-buffered saline with Tween-20 (TBST) buffer (10 mM Tris-HCl (pH 8.0), 150 mM NaCl, and 0.1% (v/v) Tween-20) at room temperature for 1 h before being probed overnight at 4 °C with anti-Panx1 (D9M1C) (#91137; Cell Signaling Technology, Beverly, MA, USA) or anti-GAPDH (GTX100118; GeneTex, Hsinchu, Taiwan) antibody diluted to 1,000 or 10,000 folds, respectively, in 5% (w/v) BSA in TBST buffer. The membrane was washed three times for 15 min each with TBST buffer and then incubated with HRP-conjugated goat anti-rabbit antibody (#GTX213110-01; GeneTex, Hsinchu, Taiwan), diluted to 3,000 (for anti-Panx1) or 10,000 (for anti-GAPDH) folds in 5% (w/v) skim milk-containing TBST buffer, for 1 h at room temperature. After a three-time wash with TBST buffer, the membrane was engulfed in a chemiluminescent substrate solution (T-Pro LumiLong Plus Chemiluminescent Substrate Kit (M), T-Pro Biotechnology, New Taipei County, Taiwan). The signals of target proteins were detected by an ImageQuant LAS 4000 Mini Biomolecular Imager (GE Healthcare Life Sciences, Milwaukee, WI, USA) using the enhanced chemiluminescence method.

### 2.17 Publicly available clinical data analysis

The mRNA expression RSEM (batch normalized from Illumina HiSeq_RNASeqV2) data were obtained from the Cancer Genome Atlas (TCGA) Lung Adenocarcinoma (LUAD) cohort via the cBioPortal for Cancer Genomics (http://www.cbioportal.org) [63,64]. The log2-transformed RSEM values from 510 LUAD patients were used for Pearson's correlation analysis between genes of interest and IL-6 via evaluating the Pearson correlation coefficient (r) and its *p*-value.

The overall survivals of lung cancer patients with low and high IL-6 or Panx1 as well as low IL-6/low Panx1 and high IL-6/high Panx1 gene expression were calculated by the Kaplan-Meier method, and their differences were analyzed using the log-rank (Mantel-Cox) test. The RNA sequencing and overall survival data of 1,087 patients derived from the GSE30219, GSE31210, GSE37745, and GSE68465 cohorts were downloaded from the Gene Expression Omnibus (GEO) database (https://www.ncbi.nlm.nih.gov/geo/). Patients in each cohort were classified into low and high IL-6 or Panx1 or low IL-6/low Panx1 and high IL-6/high Panx1 mRNA expression categories based on the median values of IL-6 and Panx1 gene expression.

### 2.18 Statistics and software

All data were analyzed with GraphPad Prism 7 software (GraphPad Inc., San Diego, CA, USA) from at least three independent experiments unless otherwise noted. The data were expressed as mean ± standard deviation (SD). The statistical tests with significant levels annotated as one asterisk (*), *p* ≤ 0.05, two asterisks (**), *p* ≤ 0.01, three asterisks (***), *p* ≤ 0.001, four asterisks (****) *p* ≤ 0.0001 or non-significance, “ns”, *p* > 0.05, were detailed in the figure legends. All figures were assembled using Adobe Illustrator CC 2020 (version v24.3.0.569) (Adobe Systems, Inc., San Jose, CA, USA).

## 3 Results

### 3.1 Sequence alignment and phylogenetic reconstruction of svLAAOs

To better understand the phylogenetic relationships between the elapid NK-LAAO and other svLAAOs, multiple protein-sequence alignments of 38 representative LAAOs in mature forms from 37 venomous snake species/subspecies and a venomous lizard served as an outgroup, retrieved from the nr database (NCBI) (Table S1), were used to infer a maximum-likelihood phylogenetic tree (Fig. 1). An important caveat is that the nature of the high guanine-cytosine (GC) content of snake genomes presents significant challenges to achieving the very high accuracy of sequencing data within the NCBI database due to the possible missing of these GC-rich regions in the assembled sequences [[65], [66], [67]]. The tree topology showed that svLAAOs were split into two major clades, clades A and B with 37 representative svLAAOs, in an early divergence event (Fig. 1). Clade A included 11 svLAAOs with 10 svLAAOs from the Elapidae family, while all 26 svLAAOs of clade B are from the Viperidae family. These separations of the enzymes remarkably correlated with the snake taxonomy except for *Cerastes Cerastes* LAAO (CC-LAAO), a viperid enzyme, but appearing in the clade of the elapid enzymes. The tree topology also indicated that the elapid NK-LAAO and NA-LAAO in clade A, sharing the most recent common ancestral sequence, are the most closely related to each other with full bootstrap support (Fig. 1, left panel).

**Fig. 1:**
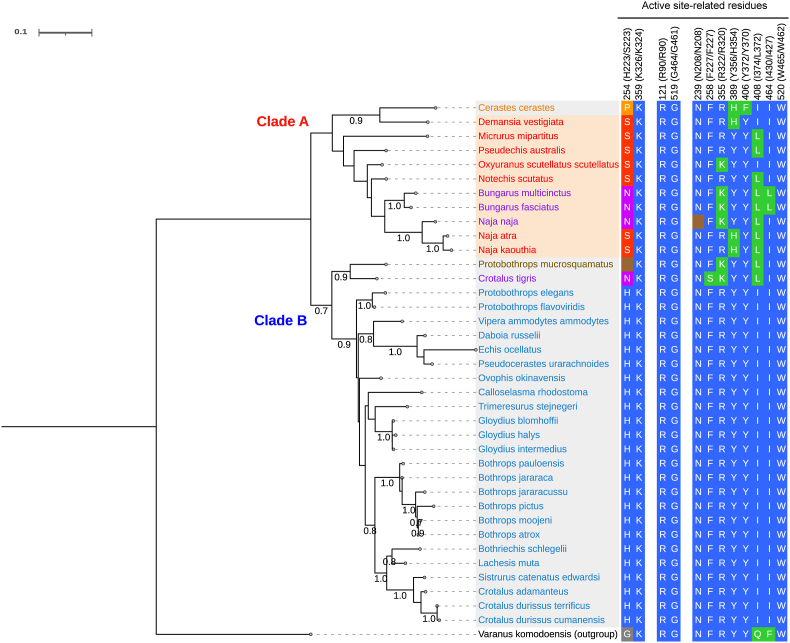
Phylogenetic tree reconstruction of svLAAOs. Left panel: A rooted maximum-likelihood (ML) phylogenetic tree reconstruction of 37 svLAAOs and an outgroup of a lizard species named *Varanus komodoensis* conducted in MEGA11 and shown by iTOL. The elapid and viperid svLAAOs were shown in light orange and light grey shadings, respectively. The ML-bootstrap values (≥0.7) calculated from 1,000 replicates were given below branches. Branch lengths were scaled to the number of amino acid substitutions per site (a scale bar of 0.1 was shown). Right panel: The active site-related residues at common positions for all svLAAOs (including gaps), or at the corresponding positions (not including gaps) in the viperid CR-LAAO and elapid NK-LAAO sequences, were obtained from multiple protein-sequence alignments of NK-LAAO with other 37 LAAOs (Table S4). These active site-related residues were considered based on the well-annotated X-ray crystallographic structures of the viperid CR-LAAO bound with citrate (CIT, PDB ID: 1F8R) or l-phenylalanine (PHE, PDB ID: 2IID). Conserved residues among svLAAOs were shown in blue shading. Substitutions or deletions at the common position 254 (position 223 without gaps) were shown in red for Ser (S), violet for Asn (N), orange for Pro (P), grey for Gly (G), or brown for deletion. Substitutions or deletions at other positions were shown in green or brown, respectively. (For interpretation of the references to color in this figure legend, the reader is referred to the Web version of this article.)

Furthermore, our analysis of multiple sequence alignments of svLAAOs (Table S4), which were used to infer the tree, revealed that most viperid svLAAOs conserved active site-related residues, including catalytic (H223 and K326) and substrate-binding (R90, G464, N208, F227, R322, Y356, Y372, I374, I430, and W465) residues, except svLAAOs from *Cerastes Cerastes* (CC-LAAO), *Protobothrop mucrusquamatus* (PM-LAAO), and *Crotalus tigris* (CT-LAAO) (Fig. 1, right panel). These active site-related residues were determined based on crystal structure studies of the viperid CR-LAAO with bound L-Phe (PHE, protein ID: 2IID) and with bound citrate (CIT, protein ID: 1F8R) [2,4]. On the contrary, elapid svLAAOs showed diverse variations of residues at these positions, especially, the substitution of His (H, blue) at position 223, a proposed critical catalytic residue present in most viperid svLAAOs, to Ser (S, red) or Asn (N, purple). The elapid NK-LAAO and NA-LAAO shared the same substitutions of Ser for His (red for blue) at position 223, His for Tyr (green for blue) at position 354 (corresponding position 356 in CR-LAAO), and Leu for Ile (green for blue) at position 372 (corresponding position 374 in CR-LAAO) (Fig. 1, right panel). Intriguingly, the elapid NK-LAAO and NA-LAAO with Ser223 presented comparable enzymatic activities on L-Leu substrate to the viperid *Bothrops atrox* LAAO (BA-LAAO) with His223 or the viperid PM-LAAO with the deletion of an amino acid at this corresponding position (Fig. S2A). Altogether, NK-LAAO is the most closely related enzyme to NA-LAAO and His223 is not always the indispensable residue for the enzymatic activity of svLAAOs.

### 3.2 Homolog modeling, molecular docking analyses, and substrate specificity of the elapid NK-LAAO

To explore the 3-D structure and catalytic mechanism, a homology model of the elapid NK-LAAO was built using an X-ray structure of the elapid NA-LAAO (PDB ID: 5Z2G), which shared the identical active site-related residues to NK-LAAO (Fig. 1), as a template. The NA-LAAO structure was determined by our group (Kumar, et al.) and is the only X-ray structure for elapid svLAAOs to date. The refined model of NK-LAAO (Fig. 2A) has a good stereochemical quality (Table S5) and can be used for further structural analysis. The NK-LAAO model is a homodimer composed of two identical protomers (Fig. 2A). In agreement with previous studies [4], each protomer (monomer) of the NK-LAAO model is composed of three distinct domains, including substrate-binding domain (SBD, green), FAD-binding domain (FBD, yellow), and helical domain (HD, violet), and a prosthetic group of non-covalently bound FAD molecule (cofactor) (Fig. 2B). These domains were assigned based on the first matching domain (5Z2G) in the CATH database [68] using NK-LAAO sequence searching. The SBD (residues 6-25, 73-129, 233-236, and 322-417) and FBD (residues 35-64, 242-316, and 443-482) contain discontinuous regions while the HD occupies residues 130-230 (Fig. 2B). In addition, each monomer was predicted to contain three putative N-linked glycosylation sites (N172, N194, and N359) (Fig. S2B). This prediction result is in line with the SDS-PAGE and MALDI-TOF MS analyses, which showed changes in protein mobility (Fig. S1F) and molecular weights (Fig. S1G) between native NK-LAAO (61.124 kDa) and Degly.NK-LAAO (59.764 kDa). Consistent with this, these three N-linked glycans were also observed in chain A of the NA-LAAO X-ray structure (5Z2G).

**Fig. 2:**
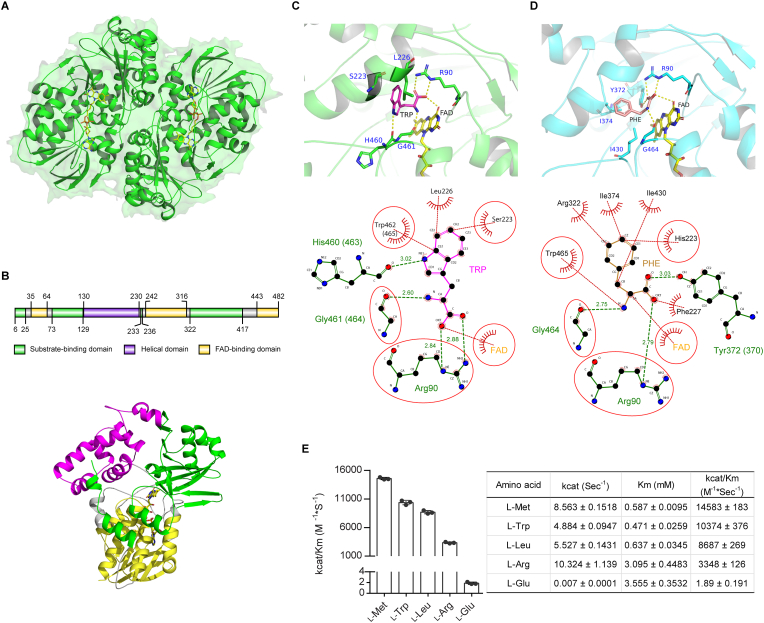
Homolog modeling, molecular docking analysis, and substrate specificity of the elapid NK-LAAO. (A) Overall structure of homolog NK-LAAO model showing surface and cartoon representations of two protomer subunits (dimer) colored in green, containing two bound FAD cofactor molecules shown in yellow stick model. (B) A protomer (monomer) showing the domain organization of NK-LAAO. Upper panel: The schematic diagram of NK-LAAO domains including the substrate-binding domain (green), FAD-binding domain (yellow), and helical domain (light violet) with their indicated boundaries. Lower panel: Cartoon representation of the three distinguishable domains and a bound FAD cofactor molecule (yellow sticks). (C, D) 3-D (upper panel, viewed by PyMOL) and 2-D (lower panel, viewed by LIGPLOT) interactions (C) between TRP (magenta stick model) and the active site-related residues of NK-LAAO in a docking model or (D) between PHE (salmon stick model) and CR-LAAO in a complex crystal structure (PDB ID: 2IID). The enlarged views of the upper panels showed the polar interactions (yellow dash lines) (C) between active site-related residues of NK-LAAO (green stick models) and TRP (magenta stick model) or (D) between CR-LAAO (cyan stick models) and PHE (salmon stick model). The lower panels showed the hydrogen bonds (green dash lines) and representative hydrophobic contacts (non-bonded contacts, red dash lines). Related residues in the NK-LAAO active site interacting with TRP and their corresponding residues in the CR-LAAO active site interacting with PHE were highlighted by red circles and ellipses. (E) Substrate specificity determined by specificity constant kcat/Km (left) and kinetic parameters (right) of NK-LAAO toward different l-amino acids (Met, Trp, Leu, Arg, and Glu) were shown. Error bars represent mean ± SD (n = 3). (For interpretation of the references to color in this figure legend, the reader is referred to the Web version of this article.)

To better understand the enzyme-substrate interactions and to find the key residues participating in the stereospecific oxidative deamination of l-amino acids by NK-LAAO, we conducted a semiflexible molecular docking of NK-LAAO (rigid) with L-Trp (TRP) (flexible). The best binding mode of the docking results showed that TRP bound at the *re*-face of the FAD isoalloxazine moiety in the NK-LAAO active site and formed polar contacts with three residues, including Arg90, His460 (corresponding position 463 in CR-LAAO), and Gly461 (corresponding position 464 in CR-LAAO), and the FAD cofactor (O4 atom) as viewed by PyMOL (Fig. 2C, upper panel). In addition, a 2-D diagram of TRP-NK-LAAO interactions generated by LIGPLOT showed that the ligand TRP made hydrogen bonds with Arg90, His460 (463), and Gly461 (464) and hydrophobic contacts with Ser223, Leu226, Trp462 (465), and the FAD cofactor (Fig. 2C, lower panel). Our results were in line with the analyses of PHE ligand complexed with CR-LAAO (Protein ID: 2IID), in which PHE made polar contacts with Arg90, Tyr372 (corresponding position 370 in NK-LAAO), Gly464, and FAD (O4 atom) (Fig. 2D, upper panel) in a 3-D schematic diagram viewed by PyMOL. Meanwhile, the 2-D schematic diagram of PHE-CR-LAAO interactions shown by LIGPLOT indicated that PHE made hydrogen bonds with Arg90, Tyr372 (370), and Gly464 and hydrophobic contacts with His223, Phe227, Arg322, Ile374, Ile430, Trp465, and FAD (Fig. 2D, lower panel). Furthermore, ligand-contacting residues in equivalent 3-D positions when NK-LAAO and CR-LAAO were superposed highlighted by red circles and ellipses include Arg90 and Gly461/464 with hydrogen bonds and Ser223/His223, Trp462/465, and FAD with hydrophobic contacts (Figs. 2C and D; lower panels).

Noticeably, kinetic studies on the substrate specificity of NK-LAAO revealed that the enzyme has a significantly high substrate preference for the hydrophobic l-amino acids L-Met, L-Trp, and L-Leu, a moderate preference for the basic l-amino acid L-Arg, and a very low specificity for the acidic l-amino acid L-Glu (Fig. 2E). Moreover, NK and NA crude venoms showed a similar pattern of LAAO enzymatic activity, which is favorable for L-Met and L-Phe but not for L-Tyr, L-Ile, L-Ala, L-Val, L-Cys, L-Gln, L-Asn, L-His, and L-Lys (Fig. S2C).

Taken together, NK-LAAO is present as homodimers with each protomer containing three domains and three putative glycosylation sites. The best binding mode of TRP in the binding pocket of NK-LAAO reveals the conservation of interactions with key residues (hydrogen bonds (Arg90 and Gly461/464) and hydrophobic contacts (Ser223/His223, Trp462/465, and FAD)), as compared to PHE in the binding pocket of CR-LAAO. Similar to other svLAAOs, NK-LAAO exhibits a high substrate preference for hydrophobic l-amino acids.

### 3.3 NK-LAAO triggers oxidative stress-mediated cell death and inhibits cell cycle progression but promotes EMT phenotype switching

In line with previous studies highlighting the anti-survival activities of several H_2_O_2_-producing svLAAOs on diverse cell types [[5], [6], [7], [8],^10^,[16], [17], [18]], our purified native elapid NK-LAAO with the sequence/structural homology and functional similarity induced substantial cytotoxic effects not only on normal non-cancerous mouse adipose-derived stromal cells (ADSCs) (Fig. S3A) and mouse adipocytes (Fig. S3B), which were differentiated from mouse ADSCs (Fig. S3C), but also on human lung adenocarcinoma A549 cells (Fig. S3D). NK-LAAO-induced H_2_O_2_ also contributed to the anti-survival activities of NK crude venom on A549 cells, as evidenced by the mitigated cytotoxic effects of NK crude venom in the presence of the H_2_O_2_-removing enzyme catalase (CAT) (Fig. S3E). Moreover, combined treatment with the highly preferred substrate L-Met, L-Trp, or L-Leu, but not with the moderately preferred L-Arg or the poorly catalyzed L-Glu, heightened the cytotoxic activities of NK-LAAO on cancer cells, whereas these effects could be counteracted by the removal of NK-LAAO-produced H_2_O_2_ with CAT (Fig. 3A). These results demonstrate that our purified native NK-LAAO possesses anti-survival activities against both normal and cancer cells with the magnitude of the cytotoxicity depending on the levels of H_2_O_2_ generated by the catalytic redox reactions of the enzyme on individual l-amino acid substrates.

**Fig. 3:**
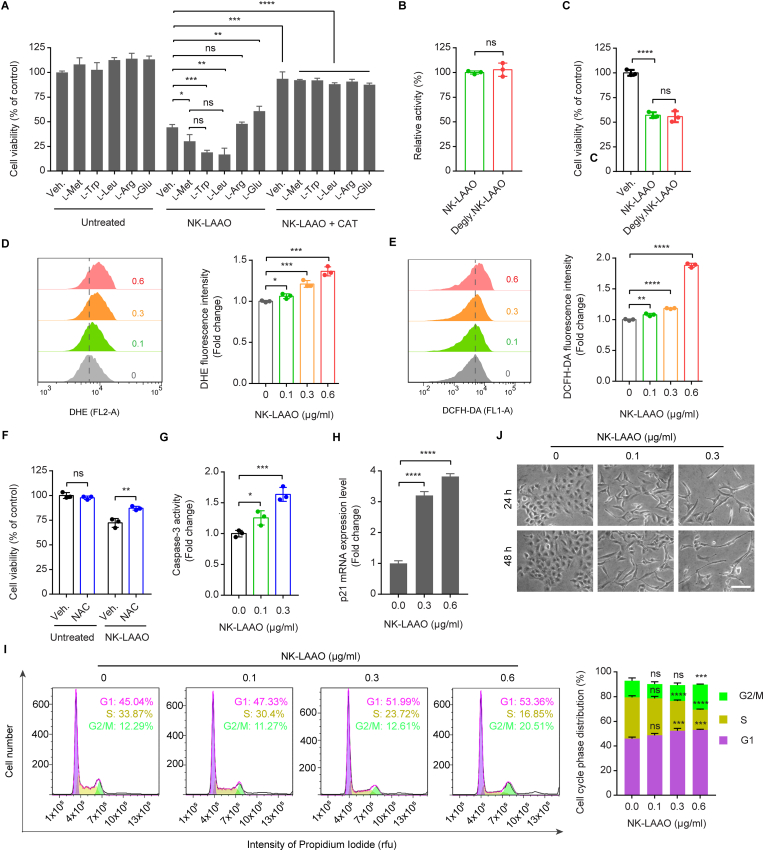
NK-LAAO triggers oxidative stress-mediated cell death and inhibits cell cycle progression but promotes EMT phenotype switching. (A) The viability of A549 cells treated with 0.6 μg/ml NK-LAAO in the presence or absence of 1 mM L-Met, L-Trp, L-Leu, L-Arg, or L-Glu alone or in combination with 400 μg/ml CAT. (B) Enzymatic activities of the native NK-LAAO and the deglycosylated NK-LAAO (Degly.NK-LAAO) on L-Leu substrate. (C) The viability of A549 cells treated with 0.6 μg/ml of NK-LAAO or Degly.NK-LAAO. Veh., Vehicle. (D, E) Flow cytometric analysis of intracellular ROS levels, stained by (D) 1 μM DHE or (E) 5 μM DCFH-DA, in A549 cells treated with increasing concentrations of NK-LAAO. (F) Cell viability was measured by MTT assay in A549 cells treated with 0.3 μg/ml NK-LAAO in the presence or absence of 0.5 mM NAC. (G) Caspase-3 activity in A549 cells treated with NK-LAAO at the indicated concentrations. (H) qRT-PCR analysis of p21 gene expression levels in A549 cells treated with the indicated concentrations of NK-LAAO. (I) Representative cell cycle histograms and quantitative measurements of cell cycle phase distributions in A549 cells treated with increasing concentrations of NK-LAAO. (J) Phase-contrast imaging of A549 cells treated with 0.1 or 0.3 μg/ml NK-LAAO. Scale bar: 100 μm. All experiments were analyzed after 24 h of NK-LAAO treatment except the phase-contrast imaging experiment (J), which was analyzed at 24 and 48 h post treatments. Error bars represent mean ± SD (n = 3). Data were analyzed using two-tailed unpaired Student's *t-*test (A–H) or two-way ANOVA (I) (*p ≤ 0.05, **p ≤ 0.01, ***p ≤ 0.001, ****p ≤ 0.0001, and ns (not significant) *p* > 0.05).

We next address the role of N-linked glycosylation in NK-LAAO functions. Strikingly, the activity of Degly.NK-LAAO toward L-Leu showed no difference compared to that of native NK-LAAO (Fig. 3B). Degly.NK-LAAO also elicited similar degrees of cytotoxicity (Figs. 3C and S3F, G) when compared to that induced by native NK-LAAO. These results suggest that both the catalytic and cytotoxic activities of NK-LAAO are independent of the N-linked glycosylation on the enzyme surface.

Exogenous H_2_O_2_ may elicit its cytotoxic effects by elevating intracellular ROS levels [69,70]. Consistently, the H_2_O_2_-producing NK-LAAO heightened intracellular ROS levels in A549 cells in a dose-dependently manner (Figs. 3D and E), and these effects could be further advanced by the addition of its highly preferred l-amino acid substrates, but not the moderately preferred or the poorly catalyzed substrates, to the culture medium (Figs. S4A and B). Furthermore, using N-acetylcysteine (NAC), a potent ROS scavenger, to abrogate NK-LAAO-elevated intracellular ROS production (Figs. S4C and D) mitigated the anti-survival effects of NK-LAAO on cancer cells (Fig. 3F). ROS can induce cell cycle arrest and apoptotic cell death [71]. Indeed, NK-LAAO as a ROS inducer augmented the activity of the ultimate executor of apoptosis caspase-3 (Fig. 3G) and the gene expression of the cyclin-dependent kinase (CDK) inhibitor p21 (Fig. 3H), which acts as a regulator to block cycle progression, leading to cell cycle inhibition (Fig. 3I). These results implicate that NK-LAAO triggers intracellular ROS production, apoptotic cell death, and cell cycle arrest. Moreover, exaggerated intracellular ROS levels induced by NK-LAAO treatment, at least in part, contribute to the cytotoxic effects of NK-LAAO on cancer cells.

H_2_O_2_-induced oxidative stress has been implicated in promoting cancer metastasis and therapeutic resistance [21,22]. We observed that NK-LAAO-treated cells acquired a tendency of morphological changes from cobblestone shape, which is typically found in untreated cells, to elongated spindle-like shape (Fig. 3J). NK-LAAO treatment also advanced the expressions of EMT-promoting (Vimentin, N-Cadherin, Snail, ZEB1, and ZEB2), antiapoptotic (Bcl-2, Bcl-xL, and Mcl-1), and antioxidant (SOD2) genes, but not housekeeping genes (GAPDH and ACTB) (Figs. S5A–K). Taken together, NK-LAAO enables cancer cells to acquire an EMT and metastatic phenotype as well as to enhance oxidative stress and apoptotic adaptability and fitness.

### 3.4 NK-LAAO-elevated IL-6 expression attenuates the sensitivity of cancer cells to NK-LAAO-induced oxidative stress

Aberrantly produced cytokines, in particular IL-6, are associated with cancer metastasis, therapeutic resistance, and poor survival outcomes in cancer patients [[25], [26], [27], [28],^30^,^72^]. A screen of gene expressions of various cytokines revealed enhanced expressions of the pro-inflammatory cytokines IL-6, IL-1β, and TNF-α in NK-LAAO-treated A549 cells as compared to untreated cells (Fig. 4A). Moreover, a similar pattern of IL-6, IL-1β, and TNF-α gene expressions was observed in cells treated with Degly.NK-LAAO (Fig. S6A), suggesting that NK-LAAO-induced pro-inflammatory cytokines production relied on its enzymatic activity but not on its anchor to the plasma membrane of cancer cells via N-linked glycans. Notably, IL-6 was the most prominent cytokine induced by NK-LAAO (Figs. 4A and S6A). NK-LAAO treatment also augmented IL-6 protein concentrations in the culture supernatants (Fig. 4B). We next examined whether the NK-LAAO-produced H_2_O_2_ and intracellular ROS participate in stimulating IL-6 expression. Exogenous H_2_O_2_ exposure increased IL-6 gene expression to levels comparable to that induced by NK-LAAO treatment (Fig. S6B). In line with the cytotoxic activities (Fig. 3A) and intracellular ROS generation (Figs. S4A and B), NK-LAAO-induced IL-6 expression could be further augmented following the combined treatment with the highly preferred l-amino acid substrates (Fig. S6C), but was diminished in the presence of the intracellular ROS scavenger NAC (Fig. 4C). Together, NK-LAAO-produced H_2_O_2_ and intracellular ROS, at least in part, contribute to IL-6 induction.

**Fig. 4:**
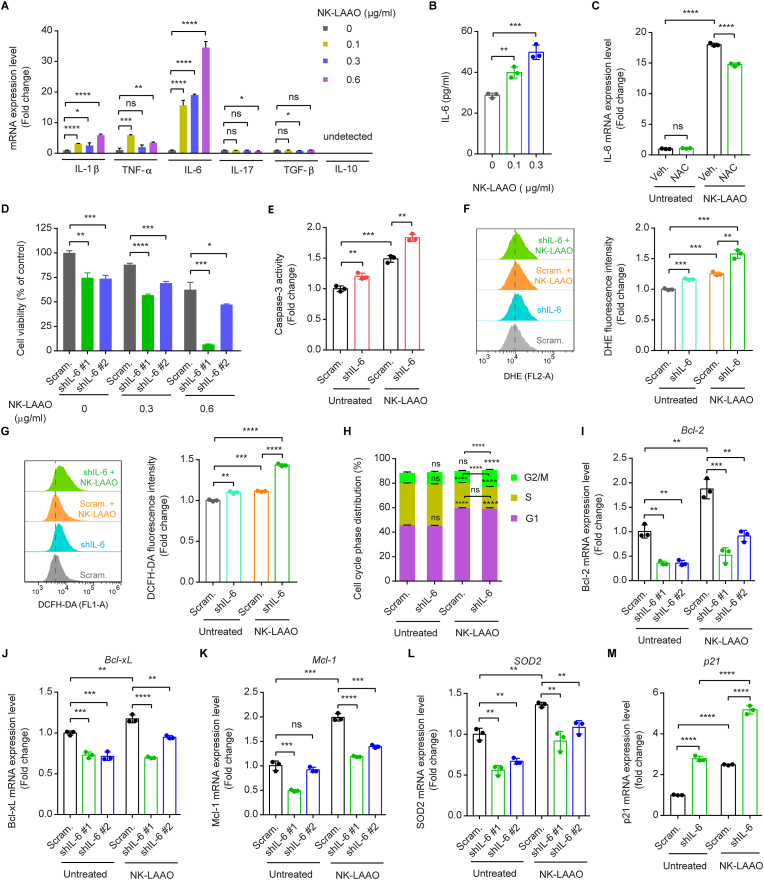
IL-6 silencing renders cells vulnerable to oxidative stress induced by NK-LAAO. (A) qRT-PCR analysis of the gene expression levels of various cytokines in A549 cells treated with increasing concentrations of NK-LAAO. (B) IL-6 protein concentrations in the culture medium of A549 cells treated with the indicated NK-LAAO concentrations were determined by ELISA. (C) qRT-PCR analysis of the IL-6 mRNA levels in A549 cells untreated or treated with 0.3 μg/ml NK-LAAO in the presence or absence of 0.5 mM NAC. (D) The viability of control (Scram.) and IL-6-silenced (shIL-6 #1 and shIL-6 #2) A549 cells untreated or treated with NK-LAAO (0.3 or 0.6 μg/ml). (E) Caspase-3 activity in control (Scram.) and IL-6-silenced (shIL-6) A549 cells untreated or treated with 0.3 μg/ml NK-LAAO. (F, G) Flow cytometric analysis of intracellular ROS levels, stained by (F) 1 μM DHE or (G) 5 μM DCFH-DA, in control and IL-6-silenced A549 cells untreated or treated with 0.3 μg/ml NK-LAAO. (H) Quantitative measurements of cell cycle phase distributions in control and IL-6-silenced A549 cells untreated or treated with 0.3 μg/ml NK-LAAO. (I–M) qRT-PCR analysis of the mRNA levels of the antiapoptotic genes (I) Bcl-2, (J) Bcl-xL, and (K) Mcl-1, (L) the antioxidant enzyme SOD2, and (M) the CDK inhibitor p21 in control and IL-6-depleted A549 cells with or without 0.3 μg/ml NK-LAAO treatment. All experiments were analyzed after 24 h of NK-LAAO treatment. Error bars represent mean ± SD (n = 3). Data were analyzed using two-tailed unpaired Student's *t-*test (A-G, I-M) or two-way ANOVA (H) (*p ≤ 0.05, **p ≤ 0.01, ***p ≤ 0.001, ****p ≤ 0.0001, and ns (not significant) *p* > 0.05).

To address the role of IL-6 in the responses of cancer cells to NK-LAAO treatment, we silenced IL-6 expression in A549 cells (Fig. S6D). IL-6 silencing significantly reduced the viability of NK-LAAO-treated cells (Fig. 4D) together with heightening NK-LAAO-induced caspase-3 activation (Fig. 4E), intracellular ROS production (Figs. 4F and G), and cell cycle arrest (Fig. 4H). Furthermore, IL-6 silencing abolished the NK-LAAO-elevated expressions of multiple genes relevant to antiapoptosis, antioxidant, and cell cycle inhibition (Figs. 4I-M). Supportedly, the IL-6 mRNA level displayed direct correlations with that of the antiapoptotic (Bcl-2 and Mcl-1) and antioxidant (SOD2) genes in the TCGA LUAD patients (Figs. S7A–C). Altogether, IL-6 confers cancer cells a robust tolerance to oxidative stress induced by NK-LAAO.

### 3.5 IL-6 silencing mitigates NK-LAAO-directed EMT phenotype switching and metastatic cell migration

We further explored the possible roles of NK-LAAO-induced IL-6 in the EMT phenotype and metastatic potential of A549 cells. IL-6 silencing reversed the morphological changes of NK-LAAO-treated cells from the prominently elongated spindle-like shape to the cobblestone shape (Fig. 5A) together with abolishing the NK-LAAO-exaggerated expressions of the EMT-stimulating genes (Figs. 5B–F). Consistently, IL-6 mRNA levels were tightly correlated with the expressions of those genes in LUAD patients (Figs. S7D–H). NK-LAAO treatment using a non-toxic condition (0.1 μg/ml for 18 h) (Fig. S8) led to approximately 1.3- and 1.2-fold increases in the migratory and invasive abilities, respectively, of cancer cells while these effects could be counteracted by IL-6 depletion (Figs. 5G and H). Taken together, IL-6 acts as a key mediator of the EMT switching and metastatic acquisition in the NK-LAAO-treated cancer cells.

**Fig. 5:**
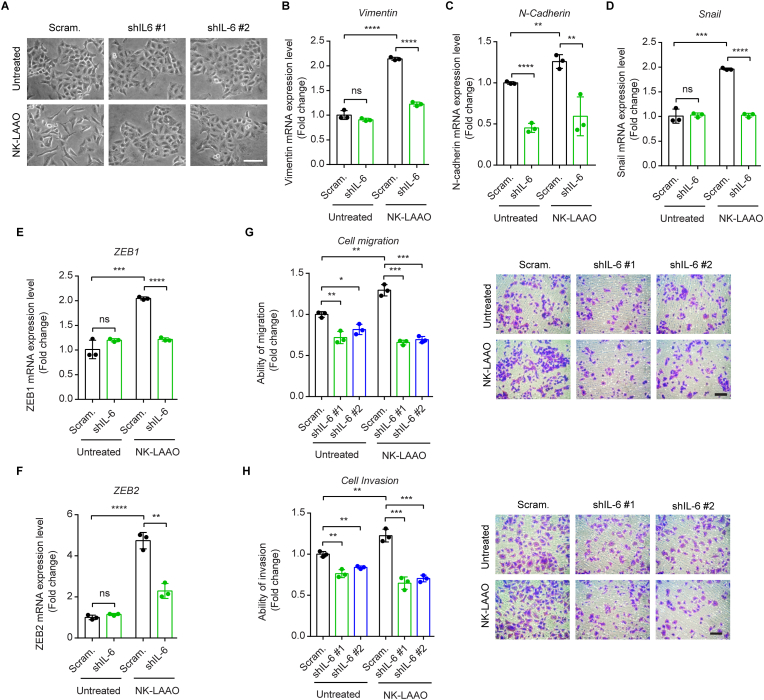
IL-6 silencing mitigates the metastatic phenotype induced by NK-LAAO. (A) Phase-contrast imaging of control and IL-6-silenced A549 cells untreated or treated with 0.3 μg/ml NK-LAAO for 24 h. Scale bar: 100 μm. (B–F) qRT-PCR analysis of the mRNA levels of the EMT inducers (B) Vimentin, (C) N-cadherin, (D) Snail, (E) ZEB1, and (F) ZEB2 in control and IL-6-silenced A549 cells with or without 0.3 μg/ml NK-LAAO treatment for 24 h. (G, H) Transwell assays monitoring the (G) migratory and (H) invasive capabilities of control and IL-6-silenced A549 cells untreated or treated with 0.3 μg/ml NK-LAAO for 18 h. Scale bar: 100 μm. Error bars represent mean ± SD (n = 3). Data were analyzed using two-tailed unpaired Student's *t-*test (*p ≤ 0.05, **p ≤ 0.01, ***p ≤ 0.001, ****p ≤ 0.0001, and ns (not significant) *p* > 0.05).

### 3.6 NK-LAAO induces IL-6 expression via the Panx1-mediated iCa^2+^ signaling pathway

Panx1 functions as a mediator of pro-inflammatory cytokines release and cancer cell metastasis and survival [39,41,73]. In agreement, Panx1 gene expression was positively correlated with the expression of IL-6 in LUAD tumor tissues (Fig. 6A). Moreover, lung cancer patients with high expressions of IL-6 (Fig. 6B), Panx1 (Fig. 6C), or both IL-6 and Panx1 (Fig. 6D) genes were associated with poorer overall survival than those having low expression levels of IL-6, Panx1, or both IL-6 and Panx1, respectively. Therefore, we hypothesized that Panx1 may be implicated in NK-LAAO-induced IL-6 expression. Indeed, ^10^Panx, a mimetic inhibitory peptide of Panx1 that blocks Panx1 activity, significantly attenuated NK-LAAO-induced IL-6 expression in a dose-dependent manner (Fig. 6E). Carbenoxolone (CBX), another widely used Panx1 inhibitor, affirmed the mediatory role of Panx1 in IL-6 expression upon NK-LAAO treatment (Fig. 6F). Moreover, similar to the phenomenon seen upon IL-6 silencing, Panx1 inhibition with ^10^Panx strengthened the cytotoxic activities of NK-LAAO against A549 cells (Fig. 6G). These findings implicate the contribution of Panx1 to NK-LAAO-driven IL-6 expression.

**Fig. 6:**
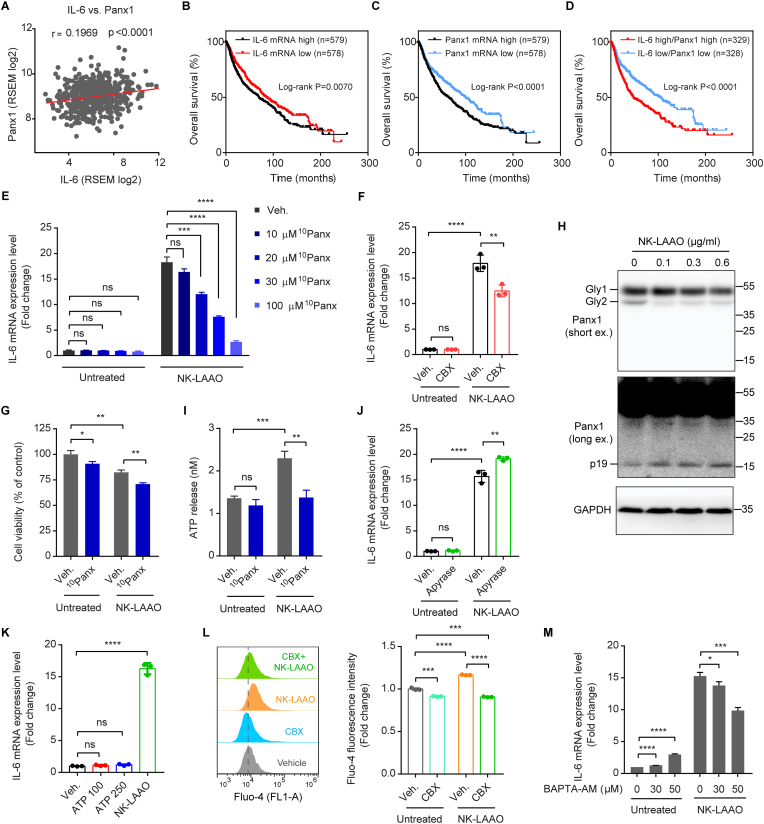
NK-LAAO induces IL-6 expression via the Panx1-mediated iCa^2+^ signaling pathway. (A) Positive correlation of mRNA expression levels between Panx1 and IL-6 in patients with lung adenocarcinoma (LUAD) (n = 510 samples). Data were extracted from The Cancer Genome Atlas (TCGA). Pearson's correlation coefficient (r) and *p*-value were shown. *p* ≤ 0.05 was considered to be statistically significant. (B–D) Kaplan-Meier analysis of the overall survival of cancer patients with low or high mRNA expression levels of (B) IL-6, (C) Panx1, or (D) IL-6 and Panx1. Data were extracted from the GSE30219, GSE31210, GSE37745, and GSE68465 cohorts derived from the Gene Expression Omnibus (GEO) database. Log-rank (Mantel-Cox) test values were shown for all comparisons, with *p* ≤ 0.05 considered to be statistically significant. (E, F) qRT-PCR analysis of the mRNA levels of IL-6 in A549 cells untreated or treated with 0.3 μg/ml NK-LAAO in the presence or absence of the Panx1 inhibitor (E) ^10^Panx (10, 20, 30, or 100 μM) or (F) CBX (50 μM) for 24 h. (G) Cell viability was measured by MTT assay in A549 cells untreated or treated with 0.3 μg/ml NK-LAAO in the presence or absence of 20 μM ^10^Panx for 24 h. (H) Western blot analysis of Panx1 protein levels in A549 cells treated with increasing concentrations of NK-LAAO for 24 h. Gly1(2), N-glycosylation species of Panx1; p19, cleaved p19 fragment of Panx1; short ex., short exposure; long ex., long exposure. (I) The extracellular ATP concentrations released by A549 cells untreated or treated with 0.3 μg/ml NK-LAAO in the presence or absence of 20 μM ^10^Panx for 24 h were measured using the luciferin-luciferase assay. (J, K) qRT-PCR analysis of the IL-6 mRNA levels in A549 cells untreated or treated with (J) 0.3 μg/ml NK-LAAO in the presence or absence of apyrase (10 U/ml) or (K) exogenous ATP (100 or 250 μM) or NK-LAAO (0.3 μg/ml) for 24 h. (L) Flow cytometric analysis of intracellular calcium levels, stained by 2 μM Fluo-4 AM, in A549 cells untreated or treated with 0.3 μg/ml NK-LAAO in the presence or absence of 50 μM CBX for 24 h. (M) qRT-PCR analysis of the IL-6 mRNA levels in A549 cells untreated or treated with 0.3 μg/ml NK-LAAO in the presence or absence of 30 or 50 μM BAPTA-AM for 24 h. Error bars represent mean ± SD (n = 3). Data were analyzed using two-tailed unpaired Student's *t-*test (E-G, I-M) (*p ≤ 0.05, **p ≤ 0.01, ***p ≤ 0.001, ****p ≤ 0.0001, and ns (not significant) *p* > 0.05).

Activated caspases (such as caspase-3) cleave the Panx1 autoinhibitory C-terminal domain to open the channel during apoptosis [32,33,35]. Concomitant with the increased activity of caspase-3 (Fig. 3G), our western blot analysis indicated the activation of Panx1 in a dose-dependent mode upon NK-LAAO treatment, as evidenced by increased levels of cleaved p19 fragments together with reduced levels of full-length Panx1 (Fig. 6H). As a result of the Panx1 opening, we detected an increase in the extracellular ATP (eATP) concentration in the culture supernatants following NK-LAAO treatment (Figs. 6I and S9A, B) but this effect was abolished in the presence of the Panx1 inhibitor ^10^Panx (Fig. 6I) or CBX (Fig. S9B). Unexpectedly, hydrolyzing extracellular ATP/ADP to AMP with the ATP diphosphohydrolase apyrase did not mitigate the levels of NK-LAAO-induced IL-6 expression (Fig. 6J). Consistent with this, treatments of cells with exogenous ATP even at very high and non-physiological concentrations (100 and 250 μM) had no significant effects on IL-6 expression (Fig. 6K). These results ruled out the possibility of extracellular ATP released via Panx1 in controlling IL-6 expression upon NK-LAAO treatment. Notably, the opening of Panx1 in NK-LAAO-treated cells led to an increase in the iCa^2+^ concentration compared to that in control cells, and this could be eliminated in the presence of the Panx1 inhibitor CBX (Fig. 6L). Furthermore, sequestering iCa^2+^ by the intracellular calcium chelator BAPTA-AM effectively hampered elevated IL-6 expression caused by NK-LAAO treatment (Fig. 6M). In conclusion, NK-LAAO-induced IL-6 expression in A549 cells is mediated, at least in part, via the Panx1/iCa^2+^ signaling pathway.

## 4 Discussion

Recently, the redox svLAAO enzymes have been proposed as promising anticancer candidates based on oxidative stress elicited by H_2_O_2_ produced during the catalytic redox reactions of the enzymes on their l-amino acid substrates [5,7,8,16,19]. However, multiple aspects of how cancer cells respond to svLAAO treatments other than being killed by svLAAOs-induced oxidative stress have not been investigated. Consistent with previous studies [[5], [6], [7],^16^,^74^], we show here that NK-LAAO possesses substantial anti-survival activities by advancing intracellular ROS levels, apoptotic cell death, and cell cycle arrest in lung adenocarcinoma A549 cells. Unexpectedly, we discover that NK-LAAO-treated cancer cells deploy a defense mechanism by amplifying the prominent expression of the pleiotropic cytokine IL-6 via the Panx1/iCa^2+^ signaling pathway to induce the expressions of antiapoptotic and antioxidant genes, which hamper the effectiveness of NK-LAAO in killing cancer cells. More importantly, NK-LAAO-produced IL-6 enables cancer cells to gain the EMT phenotype and metastatic potentials. Accordingly, it is reasonable to raise concerns regarding the potential uses of svLAAOs as well as other redox enzymes for cancer treatment.

Oxidative stress plays a dual role in cancer treatment [21,22,71,75]. In the current study, we show that oxidative stress induced by H_2_O_2_ is a major cause of NK-LAAO-mediated cell death (Fig. 3A). Our observations are in agreement with a recent study indicating that cell death induced by a recombinant svLAAO (*Naja naja* LAAO) could be entirely prevented by the addition of the H_2_O_2_-removing enzyme CAT [76]. NK-LAAO-elevated intracellular ROS levels also contribute to the anti-survival effect of NK-LAAO (Fig. 3F). However, while NAC completely abolishes advanced intracellular ROS levels produced by NK-LAAO (Figs. S4C and D), it only induces a minor increase in the viability of NK-LAAO-treated cells (Fig. 3F). This result may be interpreted by a previous suggestion that H_2_O_2_ accumulation appears to trigger cancer cell death via the direct damaging of the cell membrane rather than via the resultant increased intracellular ROS levels [77]. Moreover, our results also elucidate a tumor-supporting effect of NK-LAAO-produced H_2_O_2_. The accumulation of H_2_O_2_ generated by the redox reaction of NK-LAAO on its substrates and the subsequent intracellular ROS elevation, at least in part, contributes to NK-LAAO-induced IL-6 expression (Figs. 4C and S6C). These findings corroborate the notion that the anticancer therapy that relied on oxidative stress in general, and svLAAO activity in particular, can be a double-edged sword in cancer treatment. In addition, we suggest that although ROS scavengers, such as CAT or NAC, can diminish elevated IL-6 levels upon svLAAO treatment, these reagents should not be utilized as a tool to target IL-6 expression as they may also hamper the cytotoxic activities of svLAAOs, which mainly rely on H_2_O_2_-induced oxidative stress.

Previous studies indicated that activated Panx1 mediates the release of ATP and other signaling mediators (such as ADP, AMP, adenosine, and UTP) into the extracellular space, where they can bind to and activate purinergic receptors (P2Xs and P2Ys) to drive inflammatory cytokine expression via iCa^2+^ waves [[78], [79], [80]]. However, our results show that NK-LAAO-induced IL-6 expression is not directed by ATP release via Panx1 channels (Figs. 6I–K). Instead, we observe that the opened Panx1 channels mediate IL-6 expression upon NK-LAAO treatment by triggering a rise in iCa^2+^ concentration (Figs. 6L and M). As degradation of ATP/ADP to AMP by apyrase aggravates but not mitigates the intensity of mounted IL-6 expression induced by NK-LAAO (Fig. 6J), we speculate that the Panx1-directed iCa^2+^ elevation may result from the direct influx of Ca^2+^ through opened Panx1 channels or be controlled via P2 receptors, whose activation is mediated by Panx1-driven release of metabolites other than ATP. These results indicate that NK-LAAO-induced IL-6 expression is regulated via the Panx1/iCa^2+^ signaling pathway in an ATP-independent manner, implicating Panx1 as a target for improvement of the effectiveness of NK-LAAO treatment.

Analyses of multiple sequence alignments of svLAAOs reveal divergent evolution of the active site-related residues, especially the residue occupying the common position 254 (corresponding position 223 without gaps), in elapid svLAAO sequences (Fig. 1 and Table S4). Meanwhile, these residues are more conserved in viperid svLAAO sequences. Although His223 residue was previously proposed to play a key role in the enzymatic activity of svLAAOs by serving as a base for abstracting a proton (H^+^) from the α-amino group of the zwitterionic l-amino acid (at neutral pH) to assist the direct hydride transfer in the enzymatic reaction [2,4], analysis of site-directed mutagenesis of the viperid DR-LAAO with the respective substitution of Ser223, Asn223, or Ala223 for His223 suggested that His223 may play a role in substrate specificity rather than in catalytic activity [11]. Moreover, *Rhodococcus opacus* LAAO (RO-LAAO) from bacteria, which harbors a deletion of the amino acid residue at the position equivalent to position 223 in the CR-LAAO sequence, shows a broad substrate range [81,82]. We observe here that the venoms of the viperid *Bothrops atrox* (with His223) and *Protobothrops mucrosquamatus* (depleted the amino acid at position 223) or those of the elapid *Naja kaouthia* and *Naja atra* (with Ser223) exhibit comparable LAAO activities toward L-Leu substrate (Fig. S2A). Accordingly, it appears that His223 may not be an indispensable residue for the enzymatic activity of svLAAOs. In addition to the proposed role of His223, another possibility for substrate deprotonation comes from the active site solvent water molecule that can accept the proton from the α-NH_3_^+^ group of the substrates if the active site environment pH is high enough (pH > *p*Ka of the substrate). This general mechanism is proposed based on the X-ray structure of an ancestral LAAO N5 (AncLAAO-N5) resurrected from six LAAOs from a bacteria genus (*Pseudoalteromonas*) [83] and considered the suggested mechanism of the corresponding d-amino acid oxidases (DAAOs) [84,85]. Furthermore, the molecular docking of NK-LAAO with TRP ligand shows that while the side chain (indole) of TRP points away, its main chain is anchored at the proximity of the isoalloxazine ring (*re*-face) of FAD via two hydrogen bonds and a salt bridge (diagrams not shown) formed between the carboxylic acid group of TRP and the guanidinium group of Arg90 as well as a third hydrogen bond formed between the α-amino group of TRP and the carbonyl oxygen atom (main chain C=O) of G461 (G464 in CR-LAAO structures) (Fig. 2C). These interactions keep the Cα-H of TRP in the closed distance (3.6 Å between Cα of TRP and N5 of FAD) and the right orientation (αH pointed forward N5 atom of FAD) to the prosthetic group FAD. Our docking result is consistent with previous studies on the X-ray crystal structures of the viperid CR-LAAO [2,4], the microbial RO-LAAO [81], and the corresponding DAAOs [84,85], which support the proposed mechanism of direct hydride transfer [86].

In the present work, we show that the N-linked glycosylation on the surface of NK-LAAO presents no significant effect on the enzymatic and cytotoxic activities (Figs. 3B, C and S3F, G) as well as the expression patterns of the pro-inflammatory cytokines IL-6, IL-1β, and TNF-α (Fig. S6A) induced by NK-LAAO. On the contrary, NK-LAAO-induced cytotoxic activities, intracellular ROS production, and IL-6 expression are further advanced upon the addition of the most favorable amino acid substrate L-Met, L-Trp, or L-Leu to the culture medium. These findings suggest that the magnitudes of NK-LAAO-induced oxidative stress, anti-survival effects, and IL-6 expression depend on the levels of H_2_O_2_ generated by the catalytic redox reactions of NK-LAAO on its specific l-amino acid substrates but not on the binding of the enzyme via N-linked glycans to the cell membrane for subsequently delivering a death signal mediated by H_2_O_2_.

## 5 Conclusions and perspectives

In this study, we reveal that the catalytic and cytotoxic activities of NK-LAAO rely on the substrate composition but not on the N-link glycans on the enzyme's surface. It is important to note that different svLAAOs present distinct orders of l-amino acid substrate preferences [76,87,88]. Meanwhile, the l-amino acid composition is varying among different tumor types and is distinguished between normal and tumor tissues or even between primary and metastasizing tumors [[89], [90], [91]]. Therefore, further studies are warranted to determine specific svLAAOs that would induce the highest levels of cytotoxic effects on a certain type of tumor. Importantly, although the elapid NK-LAAO exhibits substantial anti-survival activity, it, on the contrary, stimulates the induction of the tumor-supporting and pro-metastatic cytokine IL-6 via the Panx1/iCa^2+^ signaling pathway, rendering cancer cells tolerant to NK-LAAO-mediated oxidative stress-induced cell death and gaining metastatic properties. The level of IL-6 produced by NK-LAAO treatment depends on the substrate composition and thereby is directly correlated with the degree of NK-LAAO-induced cytotoxicity. Accordingly, our findings suggest that targeting the Panx1/iCa^2+^/IL-6 signaling pathway may improve the effectiveness of svLAAOs-based anticancer therapeutics.

## Author contributions

WGW and NVT conceived the study; NVT designed the experiments, performed phylogenetic analysis, NK-LAAO structure modeling, and molecular docking analyses, conducted most of the experiments, and analyzed data; TTTP contributed to the experiments using A549 cells; TSH generated IL-6 shRNA lentiviral particles and provided technical assistance on the generation of IL-6 knockdown cells; PPD assisted with the removal of N-linked glycans on NK-LAAO. WGW and LYL supervised the study; NVT wrote the manuscript; and WGW, LYL, and NVT reviewed and revised the manuscript.

## Data availability

All data generated or analyzed during this study are available from the corresponding authors upon reasonable request.

## Declaration of competing interest

The authors declare that they have no known competing financial interests or personal relationships that could have appeared to influence the work reported in this paper.

## Figures and Tables

**figs1:**
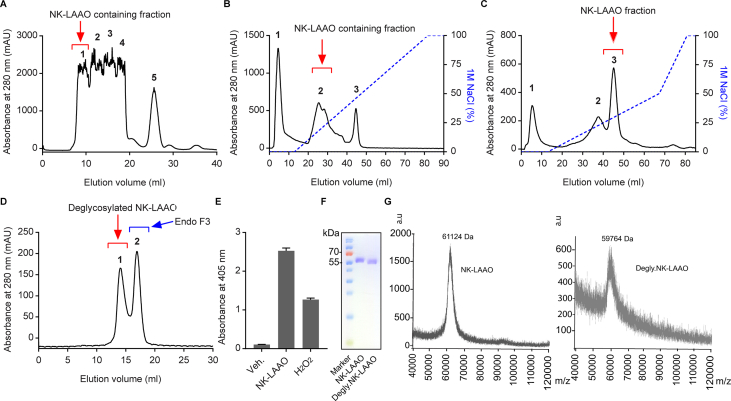
Native and deglycosylated NK-LAAO purifications.

**figs2:**
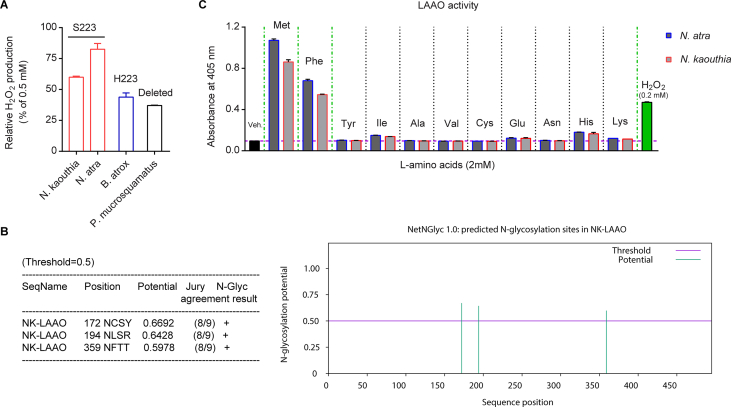
LAAO activity of crude venoms and predicted N-linked glycosylation sites in NK-LAAO.

**figs3:**
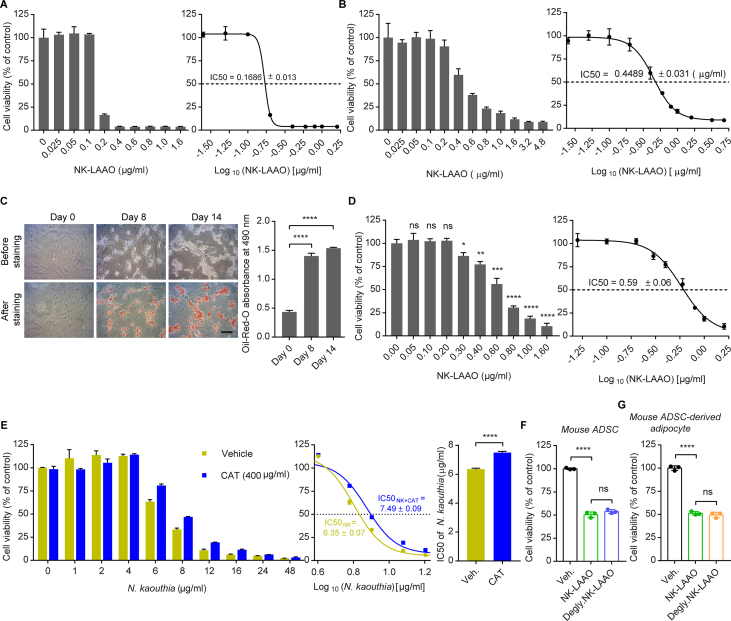
NK-LAAO possesses anti-survival effects against various cell types and its cytotoxic activity is independent of N-linked glycans on the enzyme surface.

**figs4:**
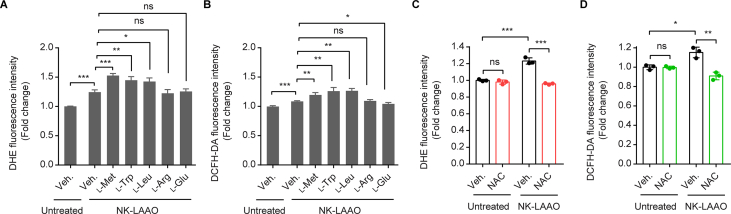
NK-LAAO-elevated intracellular ROS levels depend on individual L-amino acid components present in the culture medium.

**figs5:**
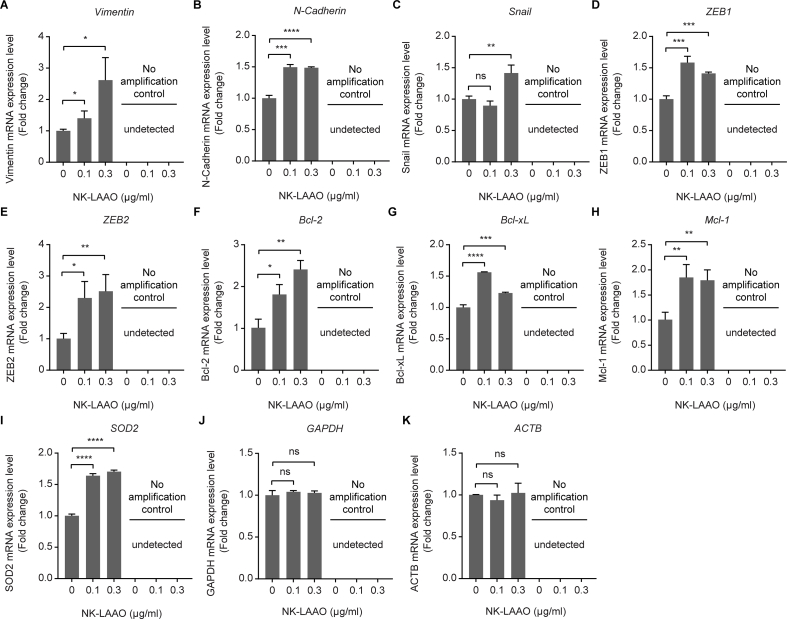
NK-LAAO treatment elevates the expressions of EMT-promoting, antiapoptotic, and antioxidant genes.

**figs6:**
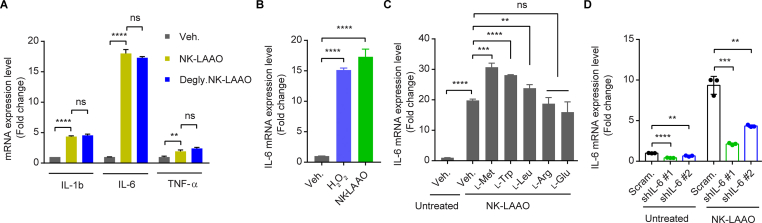
NK-LAAO-elevated IL-6 expression levels rely on individual L-amino acid components present in the culture medium but not on N-linked glycans on the enzyme’s surface.

**figs7:**
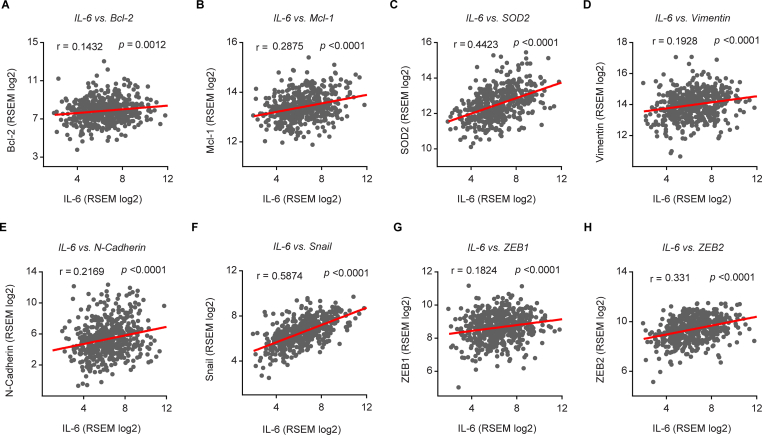
IL-6 gene expression is tightly correlated with the transcription of antiapoptotic, antioxidant, and EMT-stimulating genes in LUAD tumors.

**figs8:**
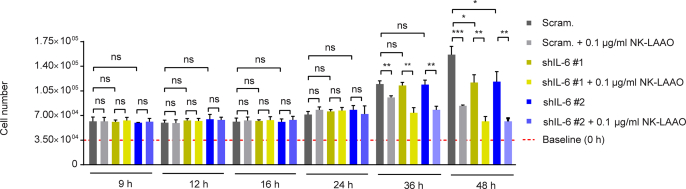
Time-dependent changes in A549 cell numbers following IL-6 knockdown in the presence or absence of NK-LAAO treatment.

**figs9:**
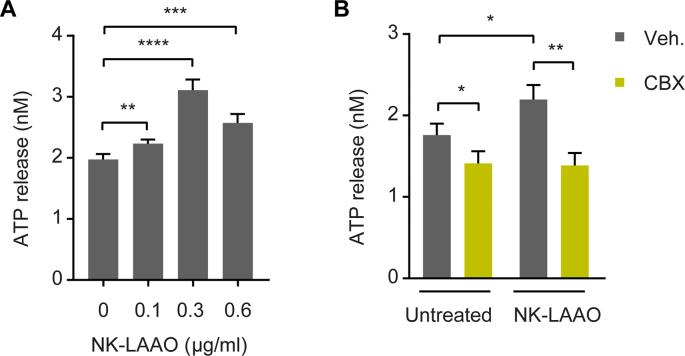
NK-LAAO treatment augments ATP release into the extracellular space via Panx1.
