# Supplementary material for: Action mechanism of snake venom l-amino acid oxidase and its double-edged sword effect on cancer treatment: Role of pannexin 1-mediated interleukin-6 expression
Source: Redox Biol. 2023 Jun 22;64:102791. doi: 10.1016/j.redox.2023.102791 (PMC10331595; doi:10.1016/j.redox.2023.102791)
Supplement: Multimedia component 1 [file mmc1.pdf]

Supplementary Information

Action mechanism of snake venom L-amino acid oxidase and its double-edged sword effect on cancer treatment: Role of pannexin 1-mediated interleukin-6 expression

Nam V. Truong[1], Trinh T. T. Phan[2], Tzu-Sheng Hsu[2], Phan Phu Duc[1], Lih-Yuan Lin[2,**], and Wen-Guey Wu[1,*]

[1]Institute of Bioinformatics and Structural Biology, College of Life Science, National Tsing Hua University, Hsinchu 300044, Taiwan R.O.C

[2]Institute of Molecular and Cellular Biology, College of Life Science, National Tsing Hua University, Hsinchu 300044, Taiwan R.O.C

* Corresponding author

** Corresponding author

E-mail addresses: lylin@life.nthu.edu.tw (L.-Y. Lin), wgwu@life.nthu.edu.tw (W.-G. Wu).

Supplementary Materials and Methods

Determination of protein concentration

Protein concentrations were determined by the bicinchoninic acid assay (BCA) using the Dual Range BCA Protein Assay Kit (Visual Protein; Energenesis Biomedical Co., Taipei, Taiwan) following the instructions of the manufacturer. The bovine serum albumin (BSA) (Thermo Scientific; Thermo Fisher Scientific Inc., Rockford, IL, USA) was used as a protein standard with the serial dilution range of 62.5-2000 μg/mL.

SDS-PAGE and MALDI-TOF MS analysis

The purity of NK-LAAO and Degly.NK-LAAO was analyzed by the sodium dodecyl sulfate-polyacrylamide gel electrophoresis (SDS-PAGE) and the matrix-assisted laser desorption/ionization-time of flight mass spectrometry (MALDI-TOF-MS) methods. The SDS-PAGE was carried out as described by Laemmli [1]. The protein ladder with a size range from 10 to 180 kDa (Thermo Scientific; Thermo Fisher Scientific Inc., Vilnius, Lithuania) was also run in parallel with the samples at the voltages of 80 and 120 for stacking (5% (w/v) polyacrylamide) and separating (12% (w/v) polyacrylamide) gels, respectively. The gel was then stained with 0.1% (w/v) Coomassie Brilliant Blue G-250 in 20% (v/v) methanol and 10% (v/v) acetic acid for 30 min and destained by a solution containing 50% (v/v) methanol and 10% (v/v) acetic acid for 3 h followed by being washed in double-distilled water (ddH$_2$O) overnight.

The molecular mass of NK-LAAO and Degly.NK-LAAO was determined by MALDI-TOF MS as described in a previous study with slight modifications [2]. Briefly, 1 μl of protein sample in ddH$_2$O containing 0.1% (v/v) trifluoroacetic acid (TFA) was spotted and air-dried on a MALDI target plate. The sample-containing spots were subsequently overlaid with 1 μl of sinapinic acid (20 mg/ml) in 50% (v/v) acetonitrile containing 0.1% (v/v) TFA. The samples were air-dried and analyzed on an

Autoflex III Smartbeam MALDI-TOF MS (Bruker Daltonik GmbH, Leipzig, Germany) with recorded spectra ranging from 40,000 to 120,000 Da.

Adipogenic induction and Oil-Red O staining

Mouse Adipose-Derived Stromal Cells (ADSCs) were cultured in the growth medium MEM α (Minimum Essential Media α) with GlutaMAX, supplemented with 10% fetal bovine serum (HyClone Laboratories, Logan, UT, USA) and 1% penicillin/streptomycin. After 24 h incubation, cells reaching 85% confluence were subjected to adipogenic differentiation. The growth medium was removed and replaced with the adipogenic differentiation medium containing the growth medium MEM α with GlutaMAX supplemented with 4 μM recombinant human insulin, 10 μM dexamethasone, 10 μM troglitazone, and 0.25 μM 3-isobutyl-1-methylxanthine. The procedure was adopted from a previous study [3]. The adipogenic differentiation medium was refreshed every 2-3 days for 14 days. To assess the formation of neutral lipid vacuoles, cells were stained with Oil-Red O at days 0, 8, and 14 as described previously [4].

Supplementary Figure Legends

Fig. S1 Native and deglycosylated NK-LAAO purifications. (A) Separation of the *Naja kaouthia* crude venom into five major fractions by the size exclusion column, Superdex 75 Increase 10/300 GL. (B) The first fraction containing NK-LAAO was then separated by the anion exchange chromatography column, HiTrap Q HP 5 ml, into three fractions under a linear gradient (indicated by blue dashed lines) from 1 to 100% (v/v) of 1 M NaCl and NK-LAAO was eluted by 25 to 30% (v/v) of 1 M NaCl in the second fraction. (C) Further separation of the second fraction by an affinity column, the HiTrap Heparin HP 5 ml, into three fractions and the purified native NK-LAAO was obtained at fraction 3 with the elution gradient from 25 to 30% (v/v) of 1 M NaCl. (D) The mixture of the purified native NK-LAAO (NK-LAAO) and the recombinant endoglycosidase F3 (endo F3), used for the removal of N-linked glycans, was separated by the Superdex 200 Increase 10/300 GL column and the deglycosylated NK-LAAO (De-gly.NK-LAAO) was collected at the first fraction. (E) Enzymatic activity of native NK-LAAO on L-Leu substrate. (F, G) The purity of isolated native NK-LAAO and De-gly.NK-LAAO was analyzed by (F) SDS-PAGE (12% (w/v) acrylamide) under reducing conditions and (G) molecular mass using the MALDI-TOF MS method. Error bars represent mean ± SD (n = 3).

Fig. S2 LAAO activity of crude venoms and predicted N-linked glycosylation sites in NK-LAAO. (A) LAAO activity of *Naja kaouthia* (*N. kaouthia*), *Naja atra* (*N. atra*), *Bothrops atrox* (*B. atrox*), and *Protobothrops mucrosquamatus* (*P. mucrosquamatus*) crude venoms on L-Leu represented by the relative amount of the H_2O_2 produced during the enzymatic reaction to 0.5 mM H_2O_2 standard. (B) Three putative N-linked glycosylation sites (N172, N194, and N359) in NK-LAAO were predicted by the NetNGlyc - 1.0 program. (C) LAAO activity of *N. kaouthia* and *N. atra* crude venoms toward 11 different L-amino acids (Met, Phe, Tyr, Ile, Ala, Val, Cys, Glu, Asn, His, and Lys). Error bars represent mean ± SD (n = 3).

Fig. S3 NK-LAAO possesses anti-survival effects against various cell types and its cytotoxic activity is independent of N-linked glycans on the enzyme surface. (A-B) The dose-dependent cytotoxic effects (left) and the dose-response curves (right) of NK-LAAO in (A) mouse ADSCs and (B) mouse ADSCs-derived adipocytes. Mouse ADSCs and ADSCs-derived adipocytes were treated with increasing concentrations of NK-LAAO and cell viability was measured by MTT assays after 24 h (the IC50 values of ADSCs and ADSCs-derived adipocytes were 0.1686 ± 0.013 μg/ml and 0.4489 ± 0.031 μg/ml, respectively). (C) Representative photomicrographs (scale bar: 100 μm) and semi-quantitative results of ADSCs stained with Oil-Red O at different time points of adipogenic induction (0, 8, and 14 days). (D) The dose-dependent cytotoxic effect (left) and the dose-response curve (right) of NK-LAAO in A549 cells. A549 cells were treated with increasing concentrations of NK-LAAO and cell viability was measured by MTT assay after 24 h (IC50 value = 0.59 ± 0.06 μg/ml). (E) The viability of A549 cells treated with *N. kaouthia* crude venom in combination with 400 μg/ml catalase (CAT, IC50 = 7.49 ± 0.09 μg/ml) or vehicle (IC50 = 6.35 ± 0.07 μg/ml) for 24 h. (F, G) The viability of (F) mouse ADSCs and (G) ADSCs-derived adipocytes treated with NK-LAAO or Degly.NK-LAAO at concentrations equivalent to their IC50. Error bars represent mean ± SD (n = 3 (A, C-G) or 6 (B)). Data were analyzed using two-tailed unpaired Student's *t*-test (**p* ≤ 0.05, ***p* ≤ 0.01, ****p* ≤ 0.001, *****p* ≤ 0.0001, and ns (not significant) *p* > 0.05).

Fig. S4 NK-LAAO-elevated intracellular ROS levels depend on individual L-amino acid components present in the culture medium. (A, B) Flow cytometric analysis of intracellular ROS levels, stained by (A) 1 μM DHE or (B) 5 μM DCFH-DA in A549 cells untreated or treated with 0.3 μg/ml NK-LAAO in the presence or absence of 1 mM L-Met, L-Trp, L-Leu, L-Arg, or L-Glu. (C, D) Flow cytometric analysis of intracellular ROS levels, stained by (C) 1 μM DHE or (D) 5 μM DCFH-DA in A549 cells untreated or treated with 0.3 μg/ml NK-LAAO in the presence or

absence of 0.5 mM NAC. All experiments were analyzed after 24 h of NK-LAAO treatment. Error bars represent mean ± SD (n = 3). Data were analyzed using two-tailed unpaired Student's *t*-test (**p* ≤ 0.05, ***p* ≤ 0.01, ****p* ≤ 0.001, and ns (not significant) *p* > 0.05).

Fig. S5 NK-LAAO treatment elevates the expressions of EMT-promoting, antiapoptotic, and antioxidant genes. qRT-PCR analysis of the expression levels of the EMT-promoting (A) Vimentin, (B) N-Cadherin, (C) Snail, (D) ZEB1, and (E) ZEB2, the antiapoptotic (F) Bcl-2, (G) Bcl-xL, and (H) Mcl-1), (I) the antioxidant SOD2, and the housekeeping (J) GAPDH and (K) ACTB genes in A549 cells treated with increasing concentrations of NK-LAAO for 24 h. Error bars represent mean ± SD (n = 3). Data were analyzed using two-tailed unpaired Student's *t*-test (**p* ≤ 0.05, ***p* ≤ 0.01, ****p* ≤ 0.001, *****p* ≤ 0.0001, and ns (not significant) *p* > 0.05).

Fig. S6 NK-LAAO-elevated IL-6 expression levels rely on individual L-amino acid components present in the culture medium but not on N-linked glycans on the enzyme's surface. (A) qRT-PCR analysis of the gene expression levels of various proinflammatory cytokines (IL-1β, IL-6, and TNFα) in A549 cells treated with 0.3 µg/ml native NK-LAAO or Degly.NK-LAAO. (B, C) qRT-PCR analysis of the IL-6 mRNA levels in A549 cells treated with (B) 0.5 mM H_2O_2 or 0.3 µg/ml NK-LAAO, or (C) 0.3 µg/ml NK-LAAO alone or in combination with 1 mM L-Met, L-Trp, L-Leu, L-Arg, or L-Glu. (D) qRT-PCR analysis of the IL-6 mRNA levels in control (Scram.) and IL-6-silenced (shIL-6 #1 and shIL-6 #2) A549 cells untreated or treated with 0.3 µg/ml NK-LAAO. All experiments were analyzed after 24 h of NK-LAAO treatment. Error bars represent mean ± SD (n = 3). Data were analyzed by two-tailed unpaired Student's *t*-test (***p* ≤ 0.01, ****p* ≤ 0.001, *****p* ≤ 0.0001, and ns (not significant) *p* > 0.05).

Fig. S7 IL-6 gene expression is tightly correlated with the transcription of antiapoptotic, antioxidant, and EMT-stimulating genes in LUAD tumors. Positive correlation of mRNA expression levels between IL-6 and the antiapoptotic (A) Bcl-2 and (B) Mcl-1, (C) the antioxidant

SOD2, and the EMT-stimulating (D) Vimentin, (E) N-Cadherin, (F) Snail, (G) ZEB1 and (H) ZEB2 genes in LUAD patients (n = 510 samples). Data were extracted from The Cancer Genome Atlas (TCGA). Pearson's correlation coefficient (r) and *p*-values were shown in each analysis and $p \leq 0.05$ was considered to be statistically significant.

Fig. S8 Time-dependent changes in A549 cell numbers following IL-6 knockdown in the presence or absence of NK-LAAO treatment. Cell counting assay monitoring the numbers of control and IL-6-depleted A549 cells with or without 0.1 μg/ml NK-LAAO treatment for the defined time periods (9, 12, 16, 24, 36, and 48 h). Error bars represent mean ± SD (n = 3). Data were analyzed by two-tailed unpaired Student's *t*-test (*$p \leq 0.05$, **$p \leq 0.01$, ***$p \leq 0.001$, and ns (not significant) $p > 0.05$).

Fig. S9 NK-LAAO treatment augments ATP release into the extracellular space via Panx1. (A) The extracellular ATP concentrations released by A549 cells treated (A) with increasing concentrations of NK-LAAO or (B) with 0.3 μg/ml NK-LAAO in the presence or absence of 50 μM CBX for 24 h were measured using the luciferin-luciferase assay. Error bars represent mean ± SD (n = 3). Data were analyzed by two-tailed unpaired Student's *t*-test (*$p \leq 0.05$, **$p \leq 0.01$, ***$p \leq 0.001$, ****$p \leq 0.0001$).

References

[1] U.K. Laemmli, Cleavage of Structural Proteins during the Assembly of the Head of Bacteriophage T4, Nature 227(5259) (1970) 680-685.

[2] Y.S. Wei, Y.R. Chang, Y.T. Tsai, Y.T. Yang, S.H. Weng, L.F. Tseng, H.C. Chou, A.T. Hu, E.C. Liao, H.Y. Chen, G.Y. Lin, W.C. Cheng, H.L. Chan, The distribution of cultivable oral anaerobic microbiota identified by MALDI-TOF MS in healthy subjects and in patients with periodontal disease, J Pharm Biomed Anal 192 (2021) 113647.

[3] S.C. Huang, T.C. Wu, H.C. Yu, M.R. Chen, C.M. Liu, W.S. Chiang, K.M. Lin, Mechanical strain modulates age-related changes in the proliferation and differentiation of mouse adipose-derived stromal cells, BMC Cell Biol 11 (2010) 18.

[4] K.J. Cho, H.E. Moon, H. Moini, L. Packer, D.Y. Yoon, A.S. Chung, Alpha-lipoic acid inhibits adipocyte differentiation by regulating pro-adipogenic transcription factors via mitogen-activated protein kinase pathways, J Biol Chem 278(37) (2003) 34823-33.
