# Supplementary material for: Action mechanism of snake venom l-amino acid oxidase and its double-edged sword effect on cancer treatment: Role of pannexin 1-mediated interleukin-6 expression
Source: Redox Biol. 2023 Jun 22;64:102791. doi: 10.1016/j.redox.2023.102791 (PMC10331595; doi:10.1016/j.redox.2023.102791)
Supplement: Multimedia component 3 [file mmc3.pdf]

**Supplementary Tables**

**Table S2. shRNA sequences**

| **shRNA name** | **Clone ID** | **Oligo sequence (5'-3')** |
|---|---|---|
| Scramble | ASN0000000004 | CCGGCCTAAGGTTAAGTCGCCCTCGCTCGAGCGA GGGCGACTTAACCTTAGGTTTTT |
| shIL-6#1 | TRCN0000059207 | CCGGCAGAACGAATTGACAAACAAACTCGAGTTT GTTTGTCAATTCGTTCTGTTTTTG |
| shIL-6#2 | TRCN0000059206 | CCGGGACATGTAACAAGAGTAACATCTCGAGATG TTACTCTTGTTACATGTCTTTTTG |

**Table S3. List of primers used for qRT-PCR**

| Name | Forward Primer (5'-3') | Reverse Primer (5'-3') |
|---|---|---|
| GAPDH | TGCACCACCAACTGCTTAGC | GGCATGGACTGTGGTCATGAG |
| ACTB (β-Actin) | CTCTTCCAGCCTTCCTTCCT | AGCACTGTGTTGGCGTACAG |
| IL-1β | CTCGCCAGTGAAATGATGGCT | GTCGGAGATTCGTAGCTGGAT |
| IL-6 | AGACAGCCACTCACCTCTTCAG | TTCTGCCAGTGCCTCTTTGCTG |
| TNF-α | TTCTGCCTGCTGCACTTTGGA | TTGATGGCAGAGAGGAGGTTG |
| IL-10 | GGAGAACCTGAAGACCCTCA | GATGTCAAACTCACTCATGGC |
| IL-17 | CTTGGAATCTCCACCGCAAT | CACGTTCCCATCAGCGTTG |
| TGF-β | GAGCCTGAGGCCGACTACTA | CGGAGCTCTGATGTGTTGAA |
| Bcl-2 | ATGTGTGTGGAGAGCGTCAA | ACAGTTCCACAAAGGCATCC |
| Bcl-xL | GAGCTGGTGGTTGACTTTCTC | TCCATCTCCGATTCAGTCCCT |
| Mcl-1 | TAAGGACAAAACGGGACTGG | ACCAGCTCCTACTCCAGCAA |
| Vimentin | GTTTCCAAGCCTGACCTCAC | GCTTCAACGGCAAAGTTCTC |
| N-Cadherin | CAACTTGCCAGAAAACTCCAGG | ATGAAACCGGGCTATCTGCTC |
| Snail | GCGAGCTGCAGGACTCTAAT | CCACTGTCCTCATCTGACA |
| ZEB1 | GCACAACCAAGTGCAGAAGA | CATTTGCAGATTGAGGCTGA |
| ZEB2 | TTCCTGGGCTACGACCATAC | TGTGCTCCATCAAGCAATTC |
| SOD2 | GTTCAATGGTGGTGGTCATATCA | GCAACTCCCCTTTGGGTTCT |
| p21 | TGTCCGTCAGAACCCATGC | AAAGTCGAAGTTCCATCGCTC |

**Table S5. Validation of NK-LAAO model**

| Server | Parameter | Before energy minimization | | After energy minimization | | Cutoff |
|---|---|---|---|---|---|---|
| PROCHECK | Most favoured regions | 802 | 93.5% | 795 | 92.7% | Goal: >90% (good quality) |
| | Additional allowed regions | 50 | 5.8% | 57 | 6.6% | |
| | Generously allowed regions | 4 | 0.5% | 4 | 0.5% | |
| | Disallowed regions | 2 | 0.2% | 2 | 0.2% | |
| | G-Factors (overall average) | -0.10 | | 0.17 | | Goal: >-0.5 (normal structures) |
| MolProbity | Poor rotamers | 13 | 1.57% | 6 | 0.73% | Goal: <0.3% |
| | Favored rotamers | 774 | 93.70% | 807 | 97.70% | Goal: >98% |
| | Ramachandran outliers | 0 | 0.00% | 2 | 0.21% | Goal: <0.05% |
| | Ramachandran favored | 916 | 96.42% | 926 | 97.47% | Goal: >98% |
| | Rama distribution Z-score | 0.27 ± 0.27 | | -0.16 ± 0.26 | | Goal: abs (Z score) <2 |
| | Cβ deviations >0.25Å | 3 | 0.33% | 0 | 0.00% | Goal: 0 |
| | Bad bonds: | 1/7884 | 0.01% | 0/7884 | 0.00% | Goal: 0% |
| | Bad angles: | 62/10668 | 0.58% | 0/10668 | 0.00% | Goal: <0.1% |

| | | | | | | |
|---|---|---|---|---|---|---|
| | Cis Prolines: | 4 / 36 | 11.11% | 4 / 36 | 11.11% | Expected: ≤1 per chain, or ≤5% |
| | CaBLAM outliers | 7 | 0.7% | 7 | 0.7% | Goal: <1.0% |
| | CA Geometry outliers | 4 | 0.42% | 4 | 0.42% | Goal: <0.5% |
| | Chiral volume outliers | 0/1164 | | 0/1164 | | |
| | Good | | Caution | | Warning | |
| **ERRAT** | Overall quality factor | 98.2851 | | 99.0374 | | |
